# Counteracting Chemoresistance with Metformin in Breast Cancers: Targeting Cancer Stem Cells

**DOI:** 10.3390/cancers12092482

**Published:** 2020-09-01

**Authors:** Samson Mathews Samuel, Elizabeth Varghese, Lenka Koklesová, Alena Líšková, Peter Kubatka, Dietrich Büsselberg

**Affiliations:** 1 Department of Physiology and Biophysics, Weill Cornell Medicine-Qatar, Education City, Qatar Foundation, Doha 24144, Qatar; elv2007@qatar-med.cornell.edu; 2 Department of Obstetrics and Gynecology, Jessenius Faculty of Medicine, Comenius University in Bratislava, 03601 Martin, Slovakia; koklesova5@uniba.sk (L.K.); liskova80@uniba.sk (A.L.); 3 Department of Medical Biology, Jessenius Faculty of Medicine, Comenius University in Bratislava, 03601 Martin, Slovakia; peter.kubatka@uniba.sk

**Keywords:** cancer, cancer stem cells, chemoresistance, metformin, multidrug resistance

## Abstract

Despite the leaps and bounds in achieving success in the management and treatment of breast cancers through surgery, chemotherapy, and radiotherapy, breast cancer remains the most frequently occurring cancer in women and the most common cause of cancer-related deaths among women. Systemic therapeutic approaches, such as chemotherapy, although beneficial in treating and curing breast cancer subjects with localized breast tumors, tend to fail in metastatic cases of the disease due to (a) an acquired resistance to the chemotherapeutic drug and (b) the development of intrinsic resistance to therapy. The existence of cancer stem cells (CSCs) plays a crucial role in both acquired and intrinsic chemoresistance. CSCs are less abundant than terminally differentiated cancer cells and confer chemoresistance through a unique altered metabolism and capability to evade the immune response system. Furthermore, CSCs possess active DNA repair systems, transporters that support multidrug resistance (MDR), advanced detoxification processes, and the ability to self-renew and differentiate into tumor progenitor cells, thereby supporting cancer invasion, metastasis, and recurrence/relapse. Hence, current research is focusing on targeting CSCs to overcome resistance and improve the efficacy of the treatment and management of breast cancer. Studies revealed that metformin (1, 1-dimethylbiguanide), a widely used anti-hyperglycemic agent, sensitizes tumor response to various chemotherapeutic drugs. Metformin selectively targets CSCs and improves the hypoxic microenvironment, suppresses the tumor metastasis and inflammation, as well as regulates the metabolic programming, induces apoptosis, and reverses epithelial–mesenchymal transition and MDR. Here, we discuss cancer (breast cancer) and chemoresistance, the molecular mechanisms of chemoresistance in breast cancers, and metformin as a chemo-sensitizing/re-sensitizing agent, with a particular focus on breast CSCs as a critical contributing factor to acquired and intrinsic chemoresistance. The review outlines the prospects and directions for a better understanding and re-purposing of metformin as an anti-cancer/chemo-sensitizing drug in the treatment of breast cancer. It intends to provide a rationale for the use of metformin as a combinatory therapy in a clinical setting.

## 1. Cancer, Chemotherapy, and Chemoresistance

Cancer remains the second leading cause of death worldwide, accounting for nearly 10 million deaths (one in six deaths is cancer-related) in 2018 alone and continues to impose an ever-growing physical, emotional, and financial burden on individuals, families, communities, and healthcare systems [1]. Nevertheless, the survival rates of several types of cancers have significantly improved due to early detection, better treatment options, and high-quality post-treatment care [1].

The foundation for ‘scientific oncology’ was laid by Dr. Giovanni Morgagni of Padua, as early as the 18th century, when the post-mortem pathologic findings from autopsies that he performed were used to relate the cause of death to the clinical course of the patient’s illness [2]. During the same period (the 1760s), almost a century before the development of anesthesia (1846), Dr. John Hunter’s (a Scottish surgeon) observation, “*If the tumor had not invaded nearby tissue and was moveable, there is no impropriety in removing it*,” paved the way for the surgical removal of solid tumors [2,3]. Over the decades and centuries, cancer treatment has drastically improved to include advanced strategies such as immunotherapy, hormone therapy, gene therapy, and stem-cell therapy in addition to precision medicine that is tailored by oncologists for patients on a case-by-case basis. However, surgical removal of the solid tumor, chemotherapy, and radiation therapy (either one, but most of the time in combinations) remains the mainstay standard in the treatment of cancer.

The depletion of the lymph nodes, bone marrow aplasia, and neutropenia observed in the autopsies performed on individuals exposed to sulfur mustard gas during first world war spurred years of research with sulfur mustard agents as a possible cure for lymphoma [4]. However, it was in the 1940s when pharmacologists Dr. Louis S. Goodman and Dr. Alfred Gilman (Yale School of Medicine, CT, USA) with the help of Dr. Gustaf Lindskog, a thoracic surgeon, injected cytotoxic ‘nitrogen mustard’ (a modified, less toxic/potent version of the mustard sulfur gas in which the sulfur was replaced by nitrogen) into a patient suffering from non-Hodgkin’s lymphoma. Later, they realized that the patient’s tumor mass significantly reduced for a few weeks after the treatment, thus thrusting into the limelight the possibility and the realization that cancer could be treated using certain chemicals/pharmacological agents [4,5]. Later in 1948, Dr. Sidney Farber (a pediatric pathologist from Harvard Medical School, MA, USA, regarded as the father of modern chemotherapy) identified that folate analogs (aminopterin and amethopterin) antagonistic to folic acid, inhibited folate requiring enzymes and caused a remission of acute lymphoblastic leukemia (ALL) in children [4,5]. Since then, either by a clear biological insight or by a pure stroke of luck, several synthetic and naturally occurring agents were identified, studied, and used in clinics for their cytotoxic and chemotherapeutic effects in the treatment of several cancers.

However, the patient responses to the chemotherapeutic drugs are often hugely variable in the clinical scenario. While some cancer subjects respond positively to drug treatment and are effectively cured of the disease, other patients show a ‘resistance’ to the drug treatment, leading to a decreased drug response and recurrence/relapse of the disease, thus contributing to over 90% of the mortality among patients with malignant tumors [6]. In some instances, cancer cells possess an inherent capacity (intrinsic/innate resistance) to survive clinically relevant dosages of the drug, resulting in inadequate initial patient response to the administered treatment [7]. Cases of ‘acquired’ resistance in which the patient shows an initial positive response to the therapeutic intervention (chemotherapy, molecular-targeted anti-cancer drugs, and cancer immunotherapy) and then gradually becomes unresponsive to the treatment as a result of acquired genetic, epigenetic, and protein alterations, leading to cancer relapse is a significant hurdle in a clinical setting [7,8]. In neoplasms, one or more complex mechanisms of tumor evolution and heterogeneity, metabolic adaptations, acquired secondary genetic and epigenetic alterations, signaling pathway feedback loops and bypass mechanisms, tumor-induced modifications of the microenvironment, and the presence of cancer stem cells (CSCs), contribute to drug resistance depending on the class of drugs and treatment strategies being employed [8,9,10]. Although our knowledge of many of the individual drug-resistance mechanisms has expanded significantly, a comprehensive understanding and means of addressing multifactorial drug-resistance mechanisms have yet to be achieved.

Among women, breast cancer remains the most prevalent type of cancer and the second leading cause of death due to cancers worldwide [11]. Breast cancers are highly heterogenous (inter-tumor and intra-tumor) in nature, and the identification of their molecular subtypes holds the key for clinicians toward the decision making with regard to the choice of the treatment strategy and prediction of the treatment outcome and prognosis [12,13,14]. In terms of the absence or presence of one or more of the estrogen receptor (ER), progesterone receptor (PR), and human epidermal growth factor receptor 2 (HER2) and the Ki-67 status, breast cancers are classified into triple-negative breast cancers (ER^−^, PR^−^, HER2^−^), luminal A (ER^+^, PR^+^, HER2^−^, Ki-67^low^), luminal B (ER^+^, PR^low^, HER2^−^, Ki-67^high^) and HER2^+^ (ER^−^, PR^−^, HER2^high/overexpressed^) subtypes [13]. While the breast cancer subtypes positive for one or more of the receptors (ER, PR, or HER2) are treated by hormonal intervention, the triple-negative breast cancers (TNBC) will not benefit from the hormone-based therapy and treatment. Using chemotherapeutic agents remains the mainstay standard for the treatment for TNBCs with very few other targeted options to control and irradicate the growth of the TNBC tumors [12,13]. The complexity among TNBCS further deepens since based on gene expression analysis and gene ontologies, TNBCs are further classified into basal-like 1 (BL1), basal-like 2 (BL2), mesenchymal (M), mesenchymal stem-like (MSL), immunomodulatory (IM), and luminal androgen receptor (LAR) subtypes [12,13,15]. In a majority of cases of breast cancer (stages I, II, and III) where the primary solid tumor is localized, the therapeutic intervention requires surgical elimination of the tumor and radiation therapy, while advanced metastatic (stage IV) breast cancers are treated using systemic drug interventions [16,17]. Furthermore, neoadjuvant/adjuvant therapy using drugs is usually administered before or after surgical removal of the tumor, respectively [16,17]. 

While modern-day diagnostics and awareness programs have increased the chances for the early detection of the disease, it is still more likely that the disease comes to light only during its later stages, since the early stages remain asymptomatic, and often, in addition to the cultural stigma that limits potential patients from seeking medical care and advice, most of them have limited access to medical care or screening programs and pose a financial burden in the absence of health insurance coverage [18,19,20]. In some instances, the efficacy of the therapeutic intervention is compromised when the patients undergoing therapy are unable to absorb the drug adequately or at times rapidly metabolize and excrete the drug, leading to less than adequate levels of the drug in the body that are required for its anti-tumor effect [21]. Additionally, especially in the elderly, the occurrence of debilitating side effects and reduced tolerance to the drug may require a reduction in the dosage to sub-optimal levels, further reducing the effect of the drug on the tumor [21]. In turn, this would require that multiple low-dose therapeutic interventions be performed to achieve maximum efficacy in the treatment, which is when the tumor cells develop resistance to the treatment. However, the most gripping concern is the fear of relapse/recurrence of the disease in patients (most of them leading ultimately to death) that adversely impact the mental health, quality of life, and adherence to post-treatment surveillance among cancer survivors who had otherwise positively responded to the various therapeutic interventions and had shown significant recovery from the disease [22,23]. 

A study that analyzed the 10-year breast cancer recurrence rates among 8062 women from the Netherlands reported that women with the HER2^+^ type of tumor had the highest rates of local recurrences (7.5%) and distant metastases (25.6%), while the luminal A subtype showed the least chance of local recurrence (3.7%) and distant metastases (9.5%) [24]. TNBC tumors, on the other hand, were characterized by high rates of regional recurrences (5.2%), while the luminal A tumors showed the lowest rates (1.7%) of regional recurrences [24]. TNBC tumors had the worst 10-year overall survival (OS) rates, while HER2^+^ tumors had the lowest 10-year recurrence-free survival (RFS) [24]. Several other studies have shown similar patterns of OS and RFS, among other populations [25,26]. More than 50% of the disease relapse/recurrence in hormone receptor-positive breast cancers occurs nearly 6+ years (recurrence rate is twice as high when compared to hormone receptor-negative tumors) after initial diagnosis and following 5 years of adjuvant endocrine therapy, posing a significant clinical challenge [27]. ER^+^ tumors have shown late recurrences, while ER-negative tumors tend to recur during the first 5 years [28]. TNBCs, on the other hand, show a high risk for distant recurrences when compared to hormone receptor-positive tumors [28]. The cancer treatment administered after initial diagnosis also significantly influences the risk of recurrence among breast cancer patients [28]. In tamoxifen-treated breast cancer patients (5-year treatment), more than 50% of all recurrence occurred between 6 and 15 years after initial diagnosis [27]. The relapse/recurrence of the disease could be mainly attributed to dormant cancer cells (undetected sub-clinical residual disease), which gain the capacity of active re-growth locally or at distant metastatic sites and the existence of ‘innate/intrinsic’ or ‘acquired’ resistance to therapeutic interventions in these cancers, where the neoplastic cells survive the primary treatment(s), resulting in the enrichment and growth of drug-resistant variant population, leading to cancer progression and relapse of the disease [21,27].

Studies have revealed that metformin (1, 1-dimethylbiguanide), a widely used anti-hyperglycemic agent, sensitizes breast tumor response to various chemo- and radiotherapeutic interventions. Metformin also selectively targets CSCs and improves the hypoxic microenvironment, suppresses the tumor metastasis and inflammation, as well as regulates the metabolic programming, induces apoptosis, and reverses epithelial–mesenchymal transition and MDR. In the current article, with particular focus on CSCs, we briefly discuss the concepts of acquired and intrinsic chemoresistance in breast cancers, lay out the possible mechanisms that contribute to breast cancer chemoresistance and the role of anti-hyperglycemic metformin as a chemo-sensitizing/re-sensitizing agent by targeting breast cancer stem cells.

## 2. Mechanisms of Chemoresistance in Breast Cancers

Cancer, once considered as a pathological condition stemming from the dysregulation of proliferation and apoptosis caused by gene mutations, has gained importance as a metabolic, inflammatory, and lifestyle disease, which can also be caused by infectious agents (bacteria and viruses) [29,30,31,32,33]. The boom in knowledge regarding the multifaceted aspects of the etiology of cancer has also revealed potential targets that can be therapeutically modified using new/re-purposed anti-cancer agents and/or radiation therapy to treat the disease efficiently. However, one of the most significant challenges faced by oncologists worldwide is the emergence of resistance to therapy, metastasis, and relapse of the disease [21]. The ‘intrinsic/innate’ resistance and, more importantly, the ‘acquired’ resistance to chemo- and radiotherapeutic interventions is a major contributor to tumor relapse/recurrence and an increase in the mortality among cancer patients [34].

**Figure 1:**
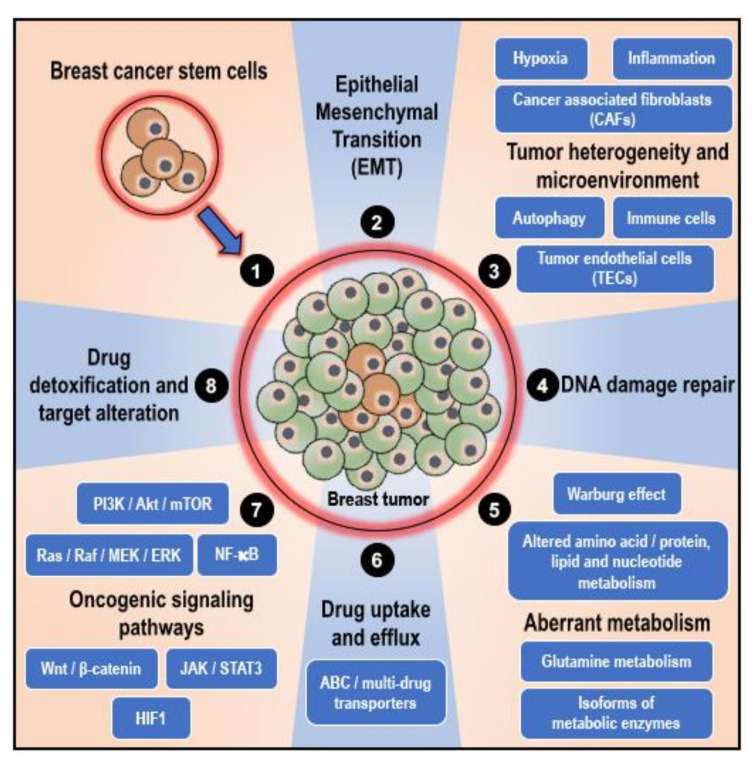
Mechanisms of chemoresistance in breast cancers: In breast cancers, the factors that influence therapeutic resistance mainly include (illustrated clockwise); (**1**) the presence and influence of breast cancer stem cells (BCSCs) that can initiate and re-populate tumors, (**2**) epithelial–mesenchymal transition (EMT), (**3**) tumor heterogeneity and microenvironment (characterized by hypoxia, inflammation, autophagy, and presence of cancer-associated fibroblasts, immune cells such as tumor-associated macrophages, and tumor endothelial cells), (**4**) active DNA damage repair mechanisms, (**5**) altered/adaptive/aberrant metabolism (characterized by the Warburg effect, altered amino acid/protein/lipid and nucleotide metabolism, utilization of glutamine, and isoforms of metabolic enzymes that support cancer initiation, progression, and resistance to therapy), (**6**) variations in drug uptake and active drug efflux systems (ATP binding cassette; ABC/multidrug transporters), (**7**) activation of oncogenic, pro-survival and anti-apoptotic signaling pathways (the phosphatidylinositol-3-kinase; PI3K/protein kinase B; Akt/ mammalian target of rapamycin; mTOR, mitogen activated protein kinase; MAPK, nuclear factor-kappa B; NF-κB, Wnt/β-catenin, janus kinase; JAK/signal transducer and activator of transcription 3; STAT3 and hypoxia inducible factor 1; HIF1 pathways), and (**8**) active drug detoxification and target alteration systems.

As mentioned earlier, at times, the positive effect of the therapeutic intervention is largely compromised when the adequate therapeutic concentration is not achieved by the patient due to reduced absorption of the drug or when the drug is metabolized and eliminated from the body [21]. Intolerance and severe side effects to therapeutic drugs also may require the administration of a reduced dosage of the drug, thus decreasing the response to the drug and overall efficacy of the treatment [21]. However, in a majority of the cases, the tumor/cancer cells themselves are genetically/epigenetically equipped to fight off/resist the cytotoxic effects of the therapeutic interventions through various resistance mechanisms that are unique, owing to the ‘intra-tumor’ and ‘inter-tumor’ heterogeneity and variability [21,35,36]. Furthermore, random variations in the expression of oncogenes and tumor suppressor genes in response to exposure to anti-cancer therapeutic interventions result in the enrichment of therapy-resistant variant cells in the tumor and the development of ‘acquired’ resistance to therapeutic intervention [21,34]. In cancers, including breast cancers, the factors that influence therapeutic resistance mainly include (Figure 1): (1) the presence of breast cancer stem cells (BCSCs), (2) epithelial–mesenchymal transition, (3) tumor heterogeneity and microenvironment, (4) active DNA damage repair mechanisms, (5) altered/adaptive/aberrant metabolism, (6) variations in drug uptake and active drug efflux systems, (7) activation of oncogenic, pro-survival, and anti-apoptotic signaling pathways, and (8) active drug detoxification and target alteration systems [21,34]. Various authoritative reviews explained these aspects in detail. As these reviews are widely available online and the topic is beyond the scope of this article, this review will not focus on these aspects [11,21,37,38,39,40,41,42,43,44,45,46]. However, similar mechanisms, in the perspective of BCSCs, which confer resistance to breast cancer against therapeutic intervention, are explained in Section 4 (role of BCSCs in chemoresistance in breast cancers) below.

## 3. Counteracting Chemoresistance in Breast Cancers: Why Metformin?

### 3.1. Indirect and ‘Direct’ Anti-Cancer/Anti-Tumor Effects of Metformin in Breast Cancers

It is a globally accepted fact that the risk of cardiovascular, hepatic, renal, and nerve diseases significantly increases in a diabetic subject [47,48,49,50,51]. In addition, the occurrence of diabetes was linked to an increase in the risk of incidence, progression, and aggressiveness of several cancers such as pancreas, liver, breast, colorectum, and skin cancers [51,52,53,54]. Furthermore, diabetes and its associated metabolic and inflammatory alterations were linked to the increasing invasiveness, metastasis, relapse/recurrence, and mortality among diabetic–cancer subjects [51,52,53,55,56,57]. The possible molecular link between diabetes and cancer (with particular reference to breast cancer) was explained in detail in our previously published article [52]. Briefly, in our previous article, we have identified the metabolic disturbances that occur in diabetes (dyslipidemia, hyperinsulinemia, and hyperglycemia) and have linked them to the downstream aberrant molecular mechanisms that play out in a cell not only leading to the incidence of breast cancer but also supporting its progression and aggressiveness and affecting the response to therapeutic interventions [52].

In light of this causal link between diabetes and breast cancer, several different classes of antidiabetic drugs were tested for their potential anti-cancer efficacy either directly or indirectly (by regulating/normalizing the blood glucose levels). Insulin and insulin analogs used in the treatment of diabetes supported the growth of the breast tumor through the activation of mitogenic and angiogenic signaling mechanisms [51,52]. However, Evans et al. in 2005, associated metformin (the most widely prescribed, well-tolerated anti-hyperglycemic drug with minimal side effects, known to be off-patent since 2002 and thus affordable) with a reduced risk/incidence of different cancers, thus leading to a spike in interest among the scientific community to study the potential anti-cancer/anti-tumor effects of metformin [51,52,58,59].

Owing to the hydrophilic and cationic nature of metformin at physiological pH, its cellular absorption, distribution, and elimination requires membrane-bound transporter molecules. While the organic cation transporters 1, 2, and 3 (OCT1, OCT2, and OCT3) support metformin transport into the cell, the plasma membrane monoamine transporter (PMAT) and multidrug and toxin extrusion protein 1 and 2 (MATE1 and MATE2) are involved in the cellular extrusion of metformin [60,61,62,63,64,65,66,67]. Reports also suggest that the intestinal absorption and renal reabsorption of metformin are dependent on the presence of the thiamine transporter 2 (THTR2), while the hepatic uptake of metformin depends on OCT1 [68,69]. Consequently, the levels of expression of the various transporters (OCTs, PMAT, and MATEs) on cancer cells would determine their sensitivity to metformin and the extent to which metformin can exert its inhibitory effect on cancer cell proliferation, invasion, and metastasis while inducing apoptotic cell death [60,61,62,63,64,65,66,67,69,70]. The overexpression of OCT3 in MCF7, BT20, and MDA-MB-468 breast cancer cells support the accumulation, and hence, the anti-proliferative effects of metformin in these cells [71]. The increased expression of OCT2 elevated the intracellular accumulation of metformin in an in vivo rat model of the mammary tumor and subsequent 5′ adenosine monophosphate-activated protein kinase (AMPK)-dependent decrease in cell proliferation, tumor volume, and suppression of tumor progression [72,73]. On the other hand, MATE2 (responsible for the extrusion of metformin from the cell) overexpression corresponded to the intrinsic resistance of several cancer cells to the anti-proliferative effect of metformin [74].

In a diabetic subject on a metformin treatment regimen, metformin controls/decreases the circulating blood glucose by decreasing (a) liver gluconeogenesis and glycogenolysis, (b) intestinal glucose absorption, and (c) the release of free fatty acids from the adipose tissue [51,75,76,77,78,79]. Additionally, metformin treatment increases cellular insulin sensitivity, leading to an increase in the rate of glucose utilization by the muscles and ultimately reduced blood glucose levels [51,75,76,77,78,79]. In turn, the decrease in the blood glucose concentration reduces the synthesis and secretion of insulin by the pancreatic β-cells and reduction in the levels of circulating insulin [51,52]. As recently published, insulin and insulin-like growth factor-1 (IGF1) activates several pro-mitogenic and pro-angiogenic signaling mechanisms and thus support pathogenesis and the progression of several cancers, including breast cancers [51,52]. Therefore, metformin treatment-related decrease in circulating insulin levels correlates to the ‘indirect’ anti-cancer/anti-tumor effects of metformin via the inhibition of the mitogenic and pro-angiogenic signaling mechanisms which are otherwise activated by insulin and IGF1 [51,52,80].

It is well-established that cancer cells (including breast cancer cells) showcase significantly altered metabolic characteristics and display a higher rate of glycolysis (Warburg effect). In cancer cells, glucose is metabolized, leading to lactate accumulation and inadequate functioning of the mitochondrial oxidative phosphorylation mechanism to rapidly derive its energy currency, adenosine triphosphate (ATP), to meet its highly demanding cellular activities to maintain cell proliferation and survival under hypoxic conditions [81]. The ability of metformin to decrease blood glucose levels and therefore reduce the availability of glucose to cancer cells accounts for the ‘direct’ anti-cancer/anti-tumor effects of metformin [51,52]. However, in the absence of glucose or when glycolysis is inhibited, cancer cells engage alternative sources and mechanisms to meet the energy requirements of the rapidly proliferating cell [82,83,84,85].

**Figure 2:**
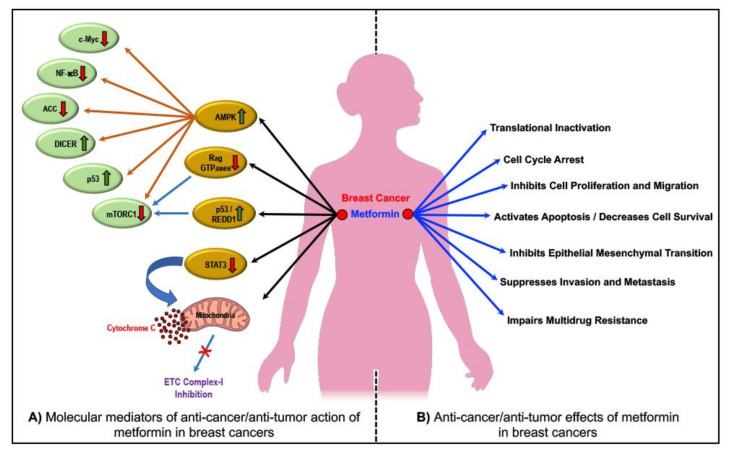
The molecular mediators and ‘direct’ anti-cancer/anti-tumor effects of metformin in breast cancers: (**A**) Metformin treatment-mediated adenosine monophosphate-activated protein kinase (AMPK) activation subsequently involves ‘AMPK-dependent’ inhibition of mTORC1, cellular-Myc (c-Myc), acetyl–CoA carboxylase (ACC), and NF-κB and pathways and/or activation of p53 pathway and double-stranded RNA specific endoribonuclease (DICER)-dependent pathways. The ‘AMPK-independent’ anti-cancer or anti-tumor effects of metformin reportedly require the activation of regulated DNA damage-1 (REDD1) and/or the inhibition of Rag GTPases and signal transducer and activator of transcription 3 (STAT3)-dependent mechanisms. (**B**) Both AMPK-dependent and independent mechanisms ultimately account for the reported in vitro, in vivo, and clinical anti-cancer effects of metformin that involve translational inactivation, cell-cycle arrest, inhibition of cellular proliferation and migration, activation of apoptotic cell death, inhibition of epithelial–mesenchymal transition (EMT), suppression of cancer invasiveness and metastasis, and counteracting multidrug/therapy resistance in breast cancers.

The ‘direct’ anti-cancer/anti-tumor effects of metformin via AMPK reportedly involve an associated downstream suppression of the mammalian target of rapamycin-C1 (mTORC1) and/or acetyl–CoA carboxylase (ACC) and/or cellular-Myc (c-Myc) and/or nuclear factor kappa-B (NF-κB) pathways or activation of the double-stranded RNA specific endoribonuclease (DICER) and/or the p53 pathways which in turn through several intracellular mediators, activation of anti-oncogenic genes, and downregulation of pro-oncogenic genes eventually accounts for the metformin treatment-related anti-proliferative, anti-migratory, pro-apoptotic and tumor-suppressive effects of metformin in cancers (Figure 2) [51,80,85,86,87,88,89,90,91,92,93,94,95,96,97,98,99,100,101]. On the other hand, metformin treatment-associated AMPK independent anti-cancer/anti-tumor/anti-proliferative and pro-apoptotic effects reportedly involve the inhibition the complex 1 of the mitochondrial electron transport chain (ETC) and/or Rag GTPases and/or signal transducer and activator of transcription-3 (STAT3) and activation of regulated DNA damage-1 (REDD1; also known as DNA damage-inducible transcript-4-DDIT4) (Figure 2) [51].

Previously, we discussed the potential of metformin as an anti-cancer drug in the treatment of breast neoplasms and described the biology and the molecular mechanism of action of metformin, in the light of the data available from various studies (in vitro, in vivo, preclinical and clinical) that have tested the potential of metformin as an anti-cancer/anti-tumor drug either as a monotherapy or in combination with other anti-cancer agents in breast cancer therapy [51]. Besides, we have identified areas that need further clarity, with regard to the anti-tumor potential of metformin, such as a therapeutic dosing of metformin specific to breast cancers, the response of breast cancer cells to metformin at high concentrations, and possible acquired resistance against metformin in all breast cancers or specific types of breast cancer. Furthermore, more studies are warranted regarding the additive or synergistic effect and efficacy of metformin when used in combination with classical anti-cancer drug treatment regimens and the possibility that metformin administration could be beneficial in breast cancer prevention in non-diabetic individuals [51].

### 3.2. Chemo-Sensitizing/Re-Sensitizing Effects of Metformin in Breast Cancer

#### 3.2.1. Endocrine Therapy Resistance and Metformin

Nearly 70% of all known breast cancers are endocrine-dependent and ER^+^ [102]. Additionally, it is known that diabetes, a key risk factor for the incidence of breast cancer, is capable of modulating estrogen availability and receptor function, thereby enhancing the risk and progression of breast cancer [52]. Although surgical removal of the tumor is involved in a majority of the breast cancer cases, adjuvant endocrine therapy has increased the overall survival while decreasing and delaying the risk of relapse of the disease [102]. Currently available adjuvant endocrine therapy includes (1) agents that block ovarian function such as goserelin (trade name: Zoladex^®^) and leuprolide (trade name: Lupron^®^), (2) drugs that block estrogen production such as the aromatase inhibitors anastrolzole (trade name: Arimidex^®^), letrozole (trade name: Femara^®^), and exemestane (trade name: Aromasin^®^), (3) selective estrogen receptor modulators (SERMs) such as tamoxifen (trade name: Nolvadex^®^) and toremifene (trade name: Fareston^®^), and (4) antiestrogenic agents such as fulvestrant (trade name: Faslodex^®^) [102].

In ER^+^ luminal A cell lines (MCF7 and ZR-75-1), while both tamoxifen and metformin treatment caused a significant dose-dependent drop in cell proliferation, a combination of tamoxifen (5 or 10 μM) and metformin (5 mM) had an additive effect on the inhibition of proliferation of the MCF7 and ZR-75-1 cells when compared to tamoxifen treatment alone [103]. The combination of metformin and tamoxifen was more effective in inhibiting the DNA replication, colony formation, and promoting apoptotic cell death of the luminal A cells when compared to cells treated with tamoxifen alone [103]. Molecular analysis revealed that combination treatment significantly mediated activation of AMPK, inhibition of the mTOR pathway, upregulation of pro-apoptotic Bax, and downregulation of anti-apoptotic Bcl2 [103]. Consistent with the in vitro data, in an in vivo nude mice model in which tumor xenografts were developed by injecting MCF7 cells into the mice, the combination treatment using metformin (2 mg/mL in drinking water) and tamoxifen (60 mg/kg by oral gavage) caused tumor growth arrest, reduction in tumor weight, and decrease in the expression/levels of Ki-67, phospho-AMPK, phospho-mTOR, and phospho-p70S6K [103].

Metformin (15–25 mM, the authors themselves have noted that these are high concentrations of metformin) treatment reportedly increased the levels of phospho-AMPK (T172) and significantly inhibited cell proliferation and the levels of the ERα protein and suppressed ERα targeted gene expression (c-Myc, cyclin D1, PR, and trefoil factor 1/TFF1/protein pS2) in both luminal A MCF7 cells and tamoxifen-resistant MCF7 cells [104]. Similar results were also obtained upon metformin treatment in estrogen-stimulated MCF7 cells and tamoxifen-resistant MCF7 cells [104]. Similar results were obtained in response to metformin treatment in luminal B MDA-MB-361 (ER^+^/HER2^+^) cells [104]. In a 1-methyl-1-nitrosourea (MNU) induced in vivo rat model of ER^+^ post-menopausal breast cancer, treatment with metformin significantly reduced tumor size while inhibiting the formation of new MNU-induced tumors [105]. The tumor microenvironment of the metformin-treated animals showed a decrease in aromatase-positive and CD68-positive macrophages and decreased lipid deposition in the liver of these animals, indicating that metformin should be capable of targeting the immune microenvironment to improve whole-body metabolism [105]. However, a phase II randomized clinical study using a combination of aromatase inhibitor (exemestane; 25 mg/day or letrozole; 2.5 mg/day) and metformin (0.5 g, twice daily, oral administration) in 60 post-menopausal women with hormone receptor-positive metastatic breast cancer failed to show an improvement in the efficacy of the endocrine therapy with the addition of metformin in the treatment regimen [106].

#### 3.2.2. Doxorubicin (DOX) and Metformin

Metformin administration-associated downregulation of platelet-derived growth factor B (PDGF-B) and subsequent inhibition of angiogenesis and the formation of immature vasculature was reported in an in vivo model of breast cancer, leading to metastatic inhibition and sensitization of the breast cancer to chemotherapeutic intervention [107]. In doxorubicin (trade name: Adriamycin^®^, DOX)-resistant breast cancer (MCF7/DOX) cells, the exposure to metformin re-sensitized the cells to DOX, which was evidenced by the significantly higher DOX-induced cytotoxicity and apoptosis in these cells after exposure to metformin [108]. This effect of metformin was correlated to the ability of metformin to the decrease in intracellular ATP content and inhibition of the activity of multidrug resistance (MDR) mediating P-glycoprotein (P-gp) [108]. The same research group further demonstrated that co-treatment of DOX and metformin, encapsulated within nanoparticles, was effective in inhibiting the activity of P-gp, leading to increased DOX uptake and an accumulation of MCF-7/DOX cells, thereby re-sensitizing the cells to DOX-induced cytotoxicity and increasing the susceptibility to apoptosis [109]. Metformin increased the tumor response to radiation in diabetic breast cancer patients and played a role in a positive response to survival [110].

Metformin (0.1 mM to 5 mM) treatment caused an AMPK activation-related suppression of cellular proliferation of parental MCF7 cells and DOX-resistant MCF7 cells in a concentration-dependent manner; however, this was to lesser extend in DOX-resistant MCF7 cells [111]. Both the DOX-sensitive and DOX-resistant MCF7 cells were sensitive to the additive anti-proliferative effect of a combinatory treatment of DOX (1 μM) and metformin (1 mM) than when either drug was used as a monotherapy [111]. Additionally, metformin treatment decreased the overexpression of multidrug-resistant (MDR) proteins in in vitro and in vivo models of DOX resistance, thereby re-sensitizing the DOX-resistant breast cancer cells and tumors to DOX treatment [111]. However, the metformin-related reduction of MDR proteins in DOX-resistant cells was independent of AMPK activity [111]. Although metformin suppressed the proliferation in both ER-positive (T47D and MCF7) and triple-negative (BT20 and MDA-MB-231) breast cancer cells, the anti-proliferative effect was higher in the ER-positive cells [111]. Davies et al. (2017), in their study, also pointed to the possibility that pre-treatment with metformin may effectively attenuate the development of drug-resistant cancers [111].

#### 3.2.3. Everolimus and Metformin

Co-treatment with metformin had an additive effect on the everolimus (an orally administered mTOR inhibitor) treatment-associated inhibition on colony formation and cell proliferation in MCF7, MDA-MB-231, and T47D breast cancer cells [112,113]. The combinatory treatment of metformin and everolimus markedly suppressed the activity of the mTOR signaling pathway and the mitochondrial respiration in these cells, although no significant changes in autophagy or apoptotic cell death were reported [112,113]. Interestingly, in the three different cells used for the study, the efficacy of metformin varied with the concentration of glucose at which the cells were maintained during the experiments. In MCF7 and MDA-MB-231 cells, metformin (5 mM) effectively reduced cell proliferation at low glucose (2.75 mM) conditions rather than at high glucose (11 mM) concentrations, while the sensitivity of T47D cells to metformin was independent of glucose concentrations [112,113].

#### 3.2.4. Trastuzumab and Metformin

Trastuzumab (brand name: Herceptin^®^) administration was effective in increasing the survival and improving outcomes in HER2-positive breast cancer patients who are otherwise associated with mostly negative outcomes and increased mortality [113,114]. Trastuzumab, a high-affinity monoclonal antibody, works by binding to the extracellular binding domain of the HER2 receptor, thereby preventing HER2 cleavage and subsequently inhibiting the cell proliferation process [113,114]. Apart from its adverse cardiotoxic effect, prolonged treatment with trastuzumab generates an acquired resistance toward the drug, giving rise to a trastuzumab-resistant highly aggressive, metastatic, and invasive subline of HER2-positive breast cancer cells [113,114,115]. The HER2/IGF-1R modulated pathway supported the increased rate of cell proliferation in the trastuzumab-resistant sublines of the cells [113,114]. Metformin treatment decreased HER2/IGF-1R activity (while HER2 expression remains unaffected) specifically in the trastuzumab-resistant forms of the HER2-overexpressing (BT474 and SKBR3) breast cancer cells. It reportedly inhibited the cellular proliferation and colony-forming capacity of the cells, while the mTOR inhibitor rapamycin failed to arrest cell growth in the resistant cell lines [114]. Furthermore, the cardioprotective effects of metformin attenuated the cardiotoxic effects of trastuzumab administration [116].

Lapatinib (trade name: Tykerb^®^), an HER2 tyrosine kinase inhibitor, is currently being used in patients with HER2 overexpressing breast carcinomas, which have shown progressive response to trastuzumab administration [113,117]. However, both intrinsic and acquired resistance was reported to compromise the efficacy of lapatinib treatment [113,117]. The hyperactivation of the phosphatidylinositol-3-kinase (PI3K)/mTOR/p70S6K1 axis was correlated to the resistance to lapatinib in lapatinib refractory models [113,117]. Metformin reportedly inhibits mTOR and subsequent p70S6K1 activity and therefore has the potential to re-sensitize lapatinib-resistant HER2 overexpressing cells to lapatinib [113,117]. Lapatinib-resistant MCF7/HER2-LapR cells were susceptible to metformin treatment, which suppressed the pathways that were linked to the acquired resistance to lapatinib in the cells [117].

The high affinity of trastuzumab for the extracellular domain of HER2 was exploited as a targeted drug delivery mechanism by linking it to emtansine, which is a drug that inhibits the microtubule assembly of the cells [118]. When the trastuzumab-emtansine (trastuzumab-DM1; T-DM1) antibody drug conjugate is administered, the trastuzumab binds to the HER2 receptors, and the internalization of T-DM1 is followed by the release of emtansine into the cells; this results in a disruption of the microtubule assembly, leading to cytotoxicity [118]. In a study of trastuzumab emtansine versus capecitabine plus lapatinib in participants with HER2^+^ locally advanced or metastatic breast cancer (EMILIA), T-DM1 treatment significantly increased progression-free survival (PFS) and overall survival (OS) in metastatic breast cancer patients [113,118]. However, the association of caveolin-1 (Cav1, a 21 kDa protein of the ‘caveolae’-cave-like structures of the membrane) and trastuzumab, facilitate the internalization of T-DM1. Thus, the efficacy of T-DM1 was dependent on the endocytotic process linked to the expression levels of Cav1 of the cells. The Cav1 expression levels (independent of the levels of HER2) widely varied between patients, and hence, T-DM1 did not work well with treatment subjects with low levels of Cav1 expression [118]. In such a scenario, a metformin treatment-induced increase in Cav1 expression enhanced T-DM1 internalization. It improved the efficacy of T-DM1 administration, causing a Cav1-linked downregulation of the Akt and MAPK pathways and promoting cell death via the reduction of pro-survival protein Bcl2 in BT474 and SKBR3 cells [118].

#### 3.2.5. Cisplatin and Metformin

Cisplatin, which is widely used in the treatment of solid tumors such as those of the breast and ovaries, reportedly binds to the reactive N7 of the purine residues in DNA, causing breaks in the DNA double-strand in rapidly dividing cancer cells and hence blocks cell division and induces cell death by apoptosis [119]. Although solid tumors consistently respond well to cisplatin intervention during the early phases of the treatment, the anti-tumor effect of cisplatin wears off over time due to the activation of the homologous recombination DNA repair mechanism, which is a form of acquired resistance to the drug [120]. RecA homolog DNA repair recombinase (RAD51) protein, the strand transferase, polymerizes into a nucleoprotein filament on single-stranded DNA. It promotes DNA strand exchange with the undamaged homologous chromatid, making it an essential component of the cellular DNA repair mechanism, the suppression of which should sensitize cancers to genotoxic DNA-damaging drugs such as cisplatin [120,121,122].

Metformin treatment (5 mM) reportedly suppressed the cisplatin-induced extracellular signal-regulated kinase (ERK)-dependent upregulation of RAD51 by decreasing the levels of phosphorylated ERK1/2, decreasing the stability of the RAD51 protein, and inducing the 26S proteasome-dependent degradation of RAD51 in Hs578T and MDA-MB-231 triple-negative breast cancer (TNBC) cells [120]. In turn, metformin enhanced the cisplatin-induced DNA damage and associated inhibitory effects on cellular proliferation, migration, and invasion and metastasis of the cells [120]. In an in vivo BALB/c mice model of breast cancer, the combination of cisplatin and metformin inhibited the increase in the volume of 4T1 cell injection-derived tumor xenografts with a correlated decrease in RAD51 levels [120].

#### 3.2.6. Multidrug Resistance and Metformin

A stepwise selection using increasing concentrations of 5-fluorouracil (5-FU) yielded MCF7 derived MCF7/5-FU population of the cells, which were resistant to multiple chemotherapeutic drugs, 5-FU, DOX (adriamycin), and paclitaxel (PTX) [123]. The MCF7/5-FU cells exhibited an altered phenotype and an inclination toward EMT [123,124,125,126]. Metformin (10 μM) treatment caused the AMPK-dependent reversal of the multidrug resistance of MCF7/5-FU cells, re-sensitized them to 5-FU, DOX, and PTX, and subsequently suppressed the EMT phenotype and inhibited the invasiveness of these cells [123]. Moreover, treatment with metformin yielded that similar results were obtained in multidrug-resistant MDA-MB-231 cells [123]. Interestingly, these effects on the breast cancer cells were shown at physiologically achievable concentrations of metformin (10 μM). A synergistic AMPK-dependent sensitization, enhancement of anti-proliferative effect, and activation of apoptosis was observed in parental MDA-MB-468, MDA-MB-231, and SKBR3 breast cancer cells when metformin (2.5 mM to 20 mM) was used in combination with 5-FU, epirubicin, and cyclophosphamide (FEC) [127].

## 4. Role of BCSCs in Chemoresistance in Breast Cancers

Pierce and Wallace, in 1971, were the first to show that in an in vivo model of squamous cell carcinoma, aggressive forms of undifferentiated cells can generate benign well-differentiated cells [128,129]. More than 20 years later (1994), a sub-population of cells distinct from the bulk tumor cells was identified in patients who had acute myeloid leukemia [130]. Then, CSCs were subsequently identified in tumors of the breast, brain, prostate, colon, and pancreas [131,132,133,134,135,136]. A mere few hundred of the cells from a small sub-population of CD44^+^/CD24^neg/low^/ESA^+^ derived from human breast cancer cells (grown in immuno-compromised non-obese diabetic/severe combined immunodeficiency; NOD/SCID mice) were capable of forming new tumors when injected into immuno-compromised NOD/SCID mice, leading to the identification of tumor-initiating/tumorigenic breast cancer cells [131]. Interestingly, the tumors generated from the tumorigenic CD44^+^/CD24^neg/low^/ESA^+^ cells also contained a diverse and mixed population of cells (tumorigenic and non-tumorigenic) that were present in the primary tumor, which is a characteristic that was indicative of the plasticity of the tumor [131]. This study laid the foundation for the concept of BCSCs in breast cancers [137]. Since the basal cells and the identified BCSCs shared similar surface markers, the study also strengthened the notion that BCSCs are derived from the normal basal mammary stem/progenitor cells as a result of mutations, transformations, and the aberrant self-renewal and differentiation pathways that occur in the basal stem/progenitor cells [131,138]. Reports have also suggested that BCSCs originate from the tumor epithelial cells that undergo EMT, since they are susceptible to transformation and share several features common to healthy and tumorigenic stem cells [139,140,141,142].

**Figure 3:**
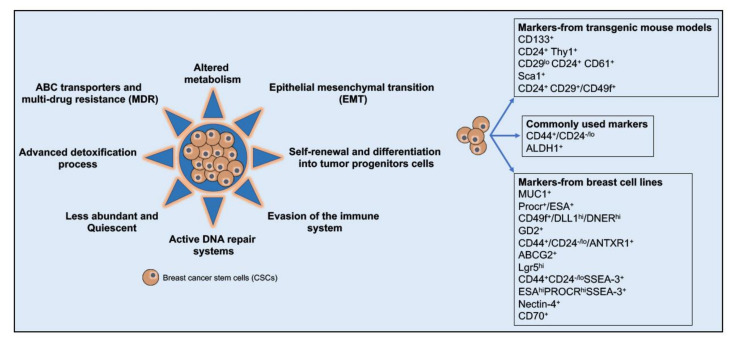
Specialized capabilities of breast cancer stem cells (BCSCs) include the power to resist therapeutic strategies: The less abundant (when compared to the terminally differentiated breast cancer cells) BCSCs with an altered metabolism are highly capable of evading therapeutic intervention and causing a relapse of the disease by employing one or more of their capabilities such as the ability to self-renew and differentiate into tumor progenitor cells, to evade the immune system, to activate unique DNA repair systems, to utilize ABC transporters for multidrug resistance (MDR) and activate advanced detoxification processes and support epithelial–mesenchymal transition (EMT). The well-studied CD44^+^/CD24^neg/low^ marker and other markers used in the identification of BCSCs are provided in the figure [157].

Chemotherapy and radiation therapy are most efficient in inhibiting cell proliferation and activating cell death and regressing the tumor in a majority of the breast neoplasms. However, reports suggest that the less abundant sub-population of tumorigenic self-renewing CSCs is enriched post-chemotherapy and radiation therapy in many different cancers, including breast cancers [143,144,145,146,147,148,149,150,151,152,153,154,155]. Thus, the BCSCs escape chemo- and radiotherapy, hampering the effectiveness of the therapeutic intervention and later supporting the invasion, metastasis, and regeneration/relapse of cancer, ultimately being responsible for the poor clinical outcomes and higher death rates among breast cancer subjects. The BCSCs, similar to all other CSCs, are endowed with specific capabilities to self-renew and differentiate, remain dormant/quiescent, and evade the immune system (Figure 3). Besides, BCSCs possess activated detoxification and advanced DNA repair mechanisms, highly expressed drug transport systems, and altered metabolism, all of which contribute to the ability of the BCSCs to intrinsically resist and escape conventional therapeutic interventions (Figure 3). Several genes regulate the stemness, self-renewal, and tumorigenic potential of BCSCs. Lower expressions of SLUG, SNAIL1, VIMENTIN and hZEB1 were observed in BCSCs, while genes such as SOX2, OCT4, NANOG, WNT, BMI1, ABCG2, ALDH1, E-CADHERIN and TWIST1 were markedly increased in the BCSCs [156].

### 4.1. BCSCs and Dormancy/Quiescence

In the bulk of the breast tumor, BCSCs make up a small sub-population of the cells with a unique ability to shift back and forth between a state of quiescence/dormancy and self-renewal and proliferation [39,138]. This enhances the survival capacity of the BCSCs when faced with adverse environmental stressors, also enabling them to evade anti-neoplastic therapeutic intervention and mask themselves from the immune system of the body [39,158,159]. The sub-population of BCSCs in the tumor are enriched after chemo- and radiation therapy, can circulate through the blood (circulating tumor cells; CTCs) and are found in pre-metastatic sites for extended periods (disseminated tumor cells; DTCs) [39,150,155,158,160]. In breast cancer subjects, slow-cycling/non-proliferating BCSCs were found (in hypoxic, necrotic, and acidic regions of the tumor) throughout the disease and played a crucial role in determining how the patient responds to therapy and ultimately the outcome of the disease [39,158]. BCSCs remain in a finely regulated quiescent state (reversible G_0_/G_1_ phase of the cell cycle), and in the event of a suitable stimulus (such as cytokine release, presence of heat-shock proteins), the BCSCs can re-enter the cell cycle. They can, self-renew and proliferate to re-populate the tumor and survive standard chemotherapeutic anti-cancer drugs, thus emphasizing their crucial role in breast cancer chemoresistance [38,39,158,160,161,162]. The persistence and survival of the quiescent /dormant BCSCs in breast tumors are linked to the hypoxic and the acidic tumor microenvironment, which in turn is linked to the resistance to therapy and less favorable patient outcomes [160,163].

### 4.2. Tumor Microenvironment

The tumor microenvironment (TME) is a decisive and emerging component that supports the plasticity and promotes the self-renewal and therapeutic resistance in BCSCs [39,43]. Cellular entities (such as endothelial cells, stromal mesenchymal cells, cancer-associated fibroblasts, immunoregulatory tumor-associated macrophages, and adipocytes), soluble and secreted factors (such as hormones, growth factors, and cytokines), the nature of the extracellular matrix, and physical parameters such as the local oxygen concentration, pH, and availability of nutrients in totality contribute to the complex nature in which the TME controls the dormancy or activation of the stem-cell-like characteristics of the BCSCs [160,161,162,163,164].

The bone marrow-derived multipotent mesenchymal stromal cells, under the influence of interleukin-6 (IL6) and chemokine (C-X-C motif) ligand-7 (CXCL7; also known as the pro-platelet basic protein), reportedly are capable of enriching the population of BCSCs in the tumor [157]. In breast cancers, activated cancer-associated fibroblasts (CAFs) regulate the BCSCs and support stem-cell-like characteristics through factors such as monocyte chemotactic protein-1 (CCL2), IL6, IL8, and high-mobility group box 1 (HMGB1) [43,157]. BCSCs control the function of CAFs, which enables the CAFs to reciprocate this by promoting the self-renewal and growth of BCSCs [37,43,157]. The macrophage colony-stimulating factors produced by the tumor cells lead to the infiltration and growth of the immunomodulatory tumor-associated macrophages (TAMs) [37,43,157]. In turn, the TAMs produce factors such as tumor necrosis factor-alpha (TNFα) and transforming growth factor-beta (TGFβ) and via the epidermal growth factor receptor (EGFR)/STAT3/sex determining region Y box transcription factor 2 (SOX2) pathway can regulate the growth and stemness of BCSCs. In addition, TAM-mediated upregulation of hyaluronan synthase 2 (HAS2) in CD44^+^/CD24^neg/low^/ESA^+^ BCSCs increases the interaction between TAMs and BCSCs [37,43,157].

The continuous supply of oxygen and nutrients in a growing tumor is achieved by vascularization of the tumor with the help of the tumor endothelial cells (TECs), which are crucial for tumor angiogenesis [43]. Apart from their crucial role in tumor angiogenesis, the TECs are capable of enriching the CD44^+^/CD24^neg/low^ BCSCs [165]. The TECs also are known to produce TNFα in response to chemotherapeutic intervention and contribute to resistance in breast cancers [43]. Targeting tumor angiogenesis and TECs, although effective as an anti-cancer treatment strategy during the initial phases of the treatment, the resultant hypoxia, due to the inhibited vascularization and restricted oxygen supply, subsequently results in persistence of BCSCs in the hypoxic environment and ultimately the rendering of the tumor resistant to chemotherapy [40,43].

The hypoxic regions within the growing breast tumors harbor the BCSCs in their quiescent/dormant states and protect them from the cytotoxic effects of the drug interventions [160]. One of the primary responses of the hypoxic tumor is the production and activation of the oxygen/hypoxia sensing hypoxia-inducible factor-1 alpha (HIF-1α) component of the HIF-1 heterodimer (made up of HIF-1α and HIF-1β) [43]. The HIF-1 transcription factor activates the expression of several drug efflux transporters such as the P-gp/ATP binding cassette subfamily B member 1 (ABCB1) by binding to the hypoxia response element on the multidrug response 1 (MDR1) gene and thereby facilitates the BCSC associated drug resistance in tumors [166]. The notch signaling mechanism and the IL-6 mediated signaling generated CD133^high^/ER^low^/IL-6^high^ BCSCs, which were capable of self-renewal and resistant to hormone-based therapy approaches [167].

### 4.3. Altered Metabolism

Under hypoxic conditions and when nutrients are scarce, the BCSCs are highly capable of adapting to such stressful circumstances by altering their metabolism [168]. The energy requirements of quiescent BCSCs are met through higher rates of glycolysis (Warburg effect), while the tumorigenic self-renewing, proliferating, and differentiating BCSCs derive their required energy from oxidative phosphorylation [168,169]. This kind of metabolic plasticity/flexibility and the ability to metabolically adapt depending on the stress in the TME help the BCSCs escape chemotherapeutic intervention and render them resistant to treatment [41]. The energy derived from the metabolic processes may be used to drive the energy-dependent efflux of the drugs using the multidrug-resistant transporters [41].

Furthermore, the lactic acid accumulation due to hyperactivated glycolysis will provide an acidic TME that is suitable to preserve the BCSCs in a quiescent state [163,170]. Various glycolytic intermediates can also be used for the synthesis of amino acids, lipids, and nucleotides, which are crucial for CSC proliferation and stemness [41]. In addition to an increased glucose uptake and increased rate of glycolysis, the available data are suggestive that glycolytic enzymes such as hexokinase, enolase, pyruvate kinase, and lactate dehydrogenase also play a central role in chemoresistance in cancers [13,41]. The metabolic plasticity and adaptations support the survival of drug-resistant phenotypes of BCSCs [13].

### 4.4. Epithelial–Mesenchymal Transition (EMT)

In breast cancers, EMT plays a crucial role in the origin of BCSCs from the tumor epithelial cells that forfeit their epithelial lineage and instead gain mesenchymal stem cell-like capacity to migrate and invade other tissues [139,140,141,142]. During EMT, the epithelial/epithelial-like cells essentially downregulate cell adhesion molecule markers such as the tight junction-associated E-cadherin (loss of E-cadherin is fundamental to the process of EMT) and cytokeratins (CK18 and CK19) while gaining the expression of mesenchymal marker N-cadherin, fibronectin, and vimentin (a cytoskeletal protein required for migration) [171,172,173,174,175]. Furthermore, the induction of EMT in breast tumors allows the newly formed BCSCs to detach from the primary tumor, resist anti-cancer treatment, and through blood circulation metastasize to distant sites [176]. Evidence also suggests that chemotherapy and radiation therapy can induce EMT in breast cancers [177,178,179]. However, the process of EMT is reversible, and upon reaching the metastatic site of localization, the cells are capable of mesenchymal–epithelial transition (MET), thus recovering their epithelial-like cell characteristics, proliferating, and populating the growing tumor [176]. The therapy-induced EMT supports the detachment of cells with stem-cell-like characteristics from primary tumors and their metastasis to other sites in the body, while MET enhances the re-population of the tumor at the secondary sites [180,181].

The quiescent CD44^+^/CD24^neg/low^ BCSCs reportedly show lower levels of E-cadherin and a higher expression of vimentin (EMT-BCSC) while proliferative; in contrast, the aldehyde dehydrogenase/ALDEFLUOR positive (ALDH^+^) BCSCs showed significantly higher levels of E-cadherin and lower expression of vimentin (MET-BCSC) [182]. Both these, the ALDH^+^ and CD44^+^/CD24^neg/low^ BCSCs, are endowed with the ability to migrate to and invade other tissues and ramp up their defense mechanisms for efficient DNA repair, detoxification, detoxification and extrusion of drugs out of the cell. As mentioned earlier, the various players involved in the TME are crucial for the plasticity of the BCSCs. Their ability to switch between epithelial–mesenchymal–epithelial states by EMT/MET renders the tumors resistant to therapeutic interventions [182]. Transcription factors such as Snail, Twist, Slug1, Zeb1, Zeb2, and FoxC2 reportedly activate EMT, which in turn is regulated by the TGFβ, Notch, Wnt, Hedgehog, NF-κB, and HIF-1 signaling pathways, all of which were implicated in a BCSC-driven therapy resistance and tumor progression [141,171,172,183].

### 4.5. Drug Efflux Transporters, Detoxification Mechanisms, and Drug Uptake-Related Chemoresistance, in BCSCs

In some instances, the effective intracellular concentration of the cytotoxic drug is not achieved in resistant cancer cells, leading to drug resistance, rather than the presence of the efflux transporters, which pump the drug out of the cell. While specific drugs enter the cell by diffusion, plasma membrane transporters are required for others. Abraxane (albumin-bound form or PTX) and DOX nanoparticle-based drugs (Doxil and Myocet) are internalized by micropinocytosis or endocytotic mechanisms (clathrin-mediated, caveolae-mediated, clathrin, and caveolin independent) [11]. While each of these mechanisms can be modified by cancer cells to limit the uptake of the drug, the clathrin and caveolin-independent endocytocic process and its modifications were implicated in providing drug uptake-based resistance to BCSCs [11,184]. The mechanisms by which differences in drug uptake by cancer cells confer resistance is a less studied area compared to studies on drug efflux/extrusion mechanisms and their role in drug resistance [11].

BCSCs are equipped with highly efficient drug efflux transporters and cellular detoxification systems that play a vital role in conferring resistance to chemotherapeutic approaches. The energy (ATP) derived from the altered metabolic processes is used to resource the ATP-dependent drug efflux transporters that maintain low levels of intracellular cytotoxic chemotherapeutic drugs by the active extrusion of these drugs from the cell. BCSCs reportedly overexpress the ATP binding cassette (ABC) transporters such as the ABCB1/multi-drug resistance protein-1 (MDR1)/P-gp, ABC sub-family G-2 (ABCG2)/breast cancer resistance protein (BCRP), ABC sub-family C-1 (ABCC1)/multidrug resistance-associated protein 1 (MRP1), ABC sub-family C-3 (ABCC3), and ATP-binding cassette sub-family B-5 (ABCB5), all of which were implicated in the ability of BCSCs to resist drug interventions and trigger cancer relapse [185]. ABCG2/BCRP and ABCB1/MDR1/P-gp that efflux Hoechst 33342 dye are also used as a function of cell surface markers for chemoresistance in BCSCs [186,187].

The overexpression of ABCG2/BCRP in a side population of MCF7-derived cells with stem cell-like features was associated with resistance to mitoxantrone (anthracycline anti-tumor agent used to treat metastatic breast cancer; substrate for ABCG2) [188]. Similar results were obtained in MDA-MB-231 TNBC cells [188]. The fine needle aspirations from recurrent metastatic breast tumors followed by dual-wavelength fluorescence activated cell sorter (FACS) analysis combined with Hoechst 33342 dye efflux revealed a sub-population of ABCG2 overexpressing adult BCSCs resistant to drug-intervention [188]. Besides, the downregulation of ABCG2 in TNBCs sensitizes the TNBCs to chemotherapeutic intervention [189]. Moreover, ABCC3 overexpression in breast cancer samples and breast cancer cell lines was associated with the increased ability of drug resistance in breast cancers [190]. While chemotherapy increased the gene expression of ABCC3, knocking down ABCC3 increased DOX retention in breast cancer cells and the sensitivity and susceptibility to the DOX and several other cytotoxic anti-neoplastic agents (mitoxantrone and methotrexate) [190].

Interestingly, knocking down ABCC3 reduced the expression of stemness-associated genes such as Nanog and a decrease in the side-population of stem-cell-like CD44^+^/CD24^neg/low^ population of the cells [190]. In an in vivo NOD/SCID mice tumor model, the inhibition/downregulation of ABCC3 was associated with increased sensitivity to DOX [190]. Similarly, ABCC1 overexpression was correlated to increased drug resistance and stemness, while the knockdown of ABCC1 reversed these effects in breast cancer cells [190].

The expression of the drug efflux transporters is regulated and modulated at multiple levels. EMT activation-associated transcription factors are major regulators of the expression of the multidrug efflux transporter expression [191,192]. Twist upregulates the expression of the drug efflux, ABC transporters, thereby contributing to chemoresistance in cancers [193]. The overexpression of SOX2 (stemness associated gene), Twist, and ABCG2 were found to be inter-related in resistance to PTX in TNBC BCSCs [156]. Disruption of the SOX2–ABCG2–Twist1 axis sensitized the TNBCs to PTX and reduced the mammosphere-forming efficiency (MSFE) and invasiveness of the cells, while the epithelial-like cell features were maintained due to the inhibition of EMT [156]. Under a hypoxic TME, the drug resistance capacity of the CSCs is enhanced by HIF1α-driven upregulation of ABCB1/MDR1 [191,192]. The NF-κB, Wnt, Notch, and Hedgehog signaling mechanisms were also implicated in the regulation of expression of the multidrug efflux transporter via EMT [191,192]. Several miRNAs additionally regulate the expression of ABC transporters and hence affect the drug resistance capabilities of the cancer cells. Tumor suppressor miR-128 sensitized breast cancers to DOX by targeting ABCC5, while the overexpression of miR-145 suppressed ABCC1/MRP1 to overcome DOX resistance in breast cancers [45,194,195]. Micro-RNAs, miR-7, and miR-326 were also implicated in the reversal of multidrug resistance in breast cancers [45,196].

Furthermore, BCSCs are characterized by an increased expression and activity of ALDH1, which is an enzyme responsible for the oxidation of intracellular aldehydes and its detoxification of carboxylic acids [139]. Both normal and cancerous breast epithelial cells gain stem-cell-like properties with an increased expression and activity of ALDH [139]. An increased expression of ALDH identifies the self-renewing tumorigenic sub-population of cells in breast carcinomas, which are capable of invasion, metastasis, and re-populating tumors at metastatic sites [139]. The high levels and activity of ALDH were directly related to the poor post-therapeutic clinical outcome on breast cancer patients [139]. The enrichment of ALDH + BCSCs was observed in breast cancer patients who underwent chemotherapy when compared to non-treated breast cancer patients [192]. Other drug detoxification systems implicated in drug resistance include dihydrofolate reductase, glutathione, and glutathione S-transferases (GST) [40]. GST reportedly is capable of inactivating cyclophosphamide, cisplatin, and DOX, which are commonly used anti-neoplastic drugs in the treatment of breast cancer [40]. The decrease in efficacy of the anti-cancer drugs was also influenced by GSTπ, which supports the detoxification and metabolism of these drugs and thereby reduces their accumulation at an effective cytotoxic concentration within the cells [40].

### 4.6. Resistance to DNA Damage: The Role of Advanced DNA Repair Systems

Despite the intricately tuned resistance mechanisms in CSCs, some cytotoxic drugs can push through these hurdles to cause DNA damage in the cells. In this worst-case scenario, the BCSCs are well equipped with advanced DNA repair mechanisms to counteract the effects of the chemotherapy and radiotherapy [197]. RAD51 mediated DNA strand exchange with the undamaged homologous chromatid, and activation of the homologous recombination DNA repair mechanism confers resistance to the TNBC against cisplatin [120]. In a mouse model of BRCA1/p53-mutated mammary tumor, clonal evolution, resistance to platinum-based chemotherapeutic agents, and enhanced tumor progression was observed in BCSC populations [198]. The interaction between the endoplasmic reticulum stress-related protein kinase RNA-like endoplasmic reticulum kinase (PERK) and nuclear erythroid-related factor 2 (NRF2) was responsible for the reduced oxidative stress, increased drug extrusion, and resistance to chemotherapy in BCSCs [199]. Moreover, the tumorigenic CD44^+^/CD24^neg/low^ side population of BCSCs derived from MCF7 and MDA-MB-231 breast cancer cells showed greater MSFE and were resistant to radiation when compared to the parental MCF7 and MDA-MB-231 breast cancer cells [200]. The radiation-induced oxidative stress, as evidenced by the levels of ROS, was significantly lesser in the stem-cell-like CD44^+^/CD24^neg/low^ cells [200]. Intermittent exposure to radiation (fractionated doses of radiation) cause a Notch-1 mediated enrichment of the tumor-initiating CD44^+^/CD24^neg/low^ side population in the MCF7 cell line [200]. Interestingly, low levels of ROS were reported in BCSCs, and they sustain less damage to the DNA when compared to the normal cells and owing to the active anti-oxidant/free radical scavenging systems that make the cells resistant to radiation therapy [201]. Pharmacological targeting of the free radical scavenging systems re-sensitized the cells to radiation [201]. Besides, increased poly (ADP-ribose) polymerase 1 (PARP1) activity and increased base excision repair, non-homologous end-joining, and BRCA1 and 2 gene mutations leading to the constitutive inactivation of homologous recombination were all implicated in DNA repair and drug resistance in BCSCs [42].

### 4.7. Signaling Pathways and BCSC-Driven Resistance

The abilities of the BCSCs to (1) self-renew, (2) switch between epithelial and mesenchymal forms, (3) adapt to the heterogenous TME, (4) maintain energy needs through altered metabolism, (5) facilitate vascular invasiveness, (6) enter into circulation and metastasize, and (7) then initiate and re-populate tumors play a crucial role in the relapse of breast tumors and increased mortality in breast cancer patients [192,202]. The BCSCs are ‘experts’ in the art of masking their identity and escaping the cytotoxic/genotoxic effects of chemo- and radiotherapeutic interventions. Similar to other healthy stem cells, all the various functions of BCSCs are tightly regulated by several signaling pathways that chiefly include (but are not limited to) the Wnt/β-catenin, the Notch, the Hedgehog (Hh), the PI3K/Akt/mTOR, the NF-κB, and the TGFβ [192,202]. ‘In-depth’ explanations on how these aberrant signaling pathways influence BCSCs to drive therapy resistance in breast cancer are beyond the scope of the current manuscript, and only the key points will be briefly discussed below while referring to and citing articles/reviews that have explained these signaling pathways in detail. Table 1 shows the role of specific pathways or factors involved in resistance against routinely used standard chemotherapeutic drugs in breast cancer.

The Wnt/β-catenin signaling in cancers is associated with the initiation of the tumor, cancer cell proliferation, migration, differentiation, survival, stemness, invasiveness, metastasis, and therapeutic resistance [12,46,192]. EMT-promoting genes such as vimentin, twist, Slug, and Snail and those that induce the expression of drug efflux transporter ABCG2/BCRP are modulated by the Wnt//β-catenin signaling mechanism [192,203,223,224]. In TNBCs, the knockdown of the β-catenin signaling was associated with a reduced stem-cell-like population, in addition to the retarded growth of the tumor and increased sensitivity to chemotherapy [225].

The activation of Notch signaling was related to stem-cell maintenance, self-renewal, differentiation, and resistance to chemotherapeutic drugs in BCSCs [12,192,226]. While Notch1-dependent upregulation of ABCC1/MRP1 and resistance to DOX was observed in T47D breast cancer cells, its inhibition was directly correlated to the sensitization of the cells to the drug [227]. Preventing the formation of the active Notch fragment, Notch intracellular domain (NICD) inhibited the proliferation and decreased the number of CD44^+^/CD24^neg/low^ and ALDH^+^ BCSC populations via the inhibition of γ-secretase and suppressed growth in in vivo tumor xenografts [192,228]. The inhibition of γ-secretase also improved the sensitivity of the cells to chemotherapeutic drugs [192,228,229]. Furthermore, the knockdown of Notch1 in DOX-resistant MCF7 cells decreased the MSFE and the number of the CD44^+^/CD24^neg/low^ BCSC population among the cells [192,230]. An increase in PTX sensitivity was observed upon Notch1 knockdown in the CD44^+^/CD24^neg/low^ population of MCF7 cells, which correlated to the downregulation of efflux transporter ABCG2/BCRP [204].

The Hedgehog (Hh) signaling pathway was associated with self-renewal capacity and tumorigenesis in cancers [12,192]. The expression of the EMT-associated gene, Snail, was upregulated by Hh signaling and glioma-associated oncogene homolog (Gli) related transcriptional activity [12,192,231,232,233]. Hh signaling mediates the expression of multidrug resistance proteins such as ABCB1/P-gp and ABCG2/BCRP [234]. Exposure to docetaxel caused a Hh signaling-associated increase in the CD44^+^/CD24^neg/low^ BCSC population and increased the MSFE [235].

The PI3K/Akt/mTOR and PTEN pathway, and its deregulation, was directly linked to cancer cell proliferation and cancer progression in many different cancers [12]. The expressions of the drug efflux transporters BCRP/ABCG2 and MDR1/ABCB1/P-gp were significantly higher in the stem-cell-like side-population of MCF7 cells that were resistant to mitoxantrone and carboplatin when compared to the parental MCF7 cells [236]. While tamoxifen effectively inhibited the cell proliferation of adherent breast cancer cells, the CSC population derived from these cells was resistant to tamoxifen treatment [237]. Exposure to tamoxifen activated the mTOR signaling mechanisms in the BCSCs, resulting in resistance to the drug, while the pharmacological inhibition of mTOR decreased the MSFE of the BCSCs and sensitized the cells to tamoxifen treatment [237]. Moreover, a markedly increased expression of HER2 and resistance to trastuzumab was observed in breast cancer cells co-cultured with mesenchymal stem cells (MSCs) [238]. Upon examination, reduced expression to a complete loss of PTEN was observed with the subsequent activation of the PI3K/Akt mechanism in the breast cancer cells co-cultured with MSCs [238].

The activation of NF-κB signaling in mammary epithelial cells plays a crucial role in mammary tumorigenesis [239]. BCSCs were associated with high NF-κB-inducing kinase (NIK) expression, while its inhibition led to the reduction of CSC markers in breast cancer cell lines [240]. The inhibition of NF-κB caused the suppression of CD44 levels in TNBCs cells and was correlated to the reduced invasiveness of these cells [241]. Besides, the hypoxia-mediated upregulation of NF-κB signaling was reported to cause resistance to chemotherapeutic drugs in cancers [12,46].

TGFβ was implicated in BCSC self-renewal/proliferation, EMT, tumor angiogenesis, invasion, metastasis, and drug resistance [46]. The upregulation/overexpression of TGFβ in breast cancer cells promoted EMT, increased stemness, and conferred drug resistance [242]. The increased expression of TGFβ and corresponding signaling correlated to the upregulation of CSC markers in epirubicin (Epi) resistance breast cancer cell lines [11].

## 5. Effect of Metformin on BCSCs in Counteracting Drug Resistance

### 5.1. Possible Mechanisms of Action of Metformin on BCSCs

**Figure 4:**
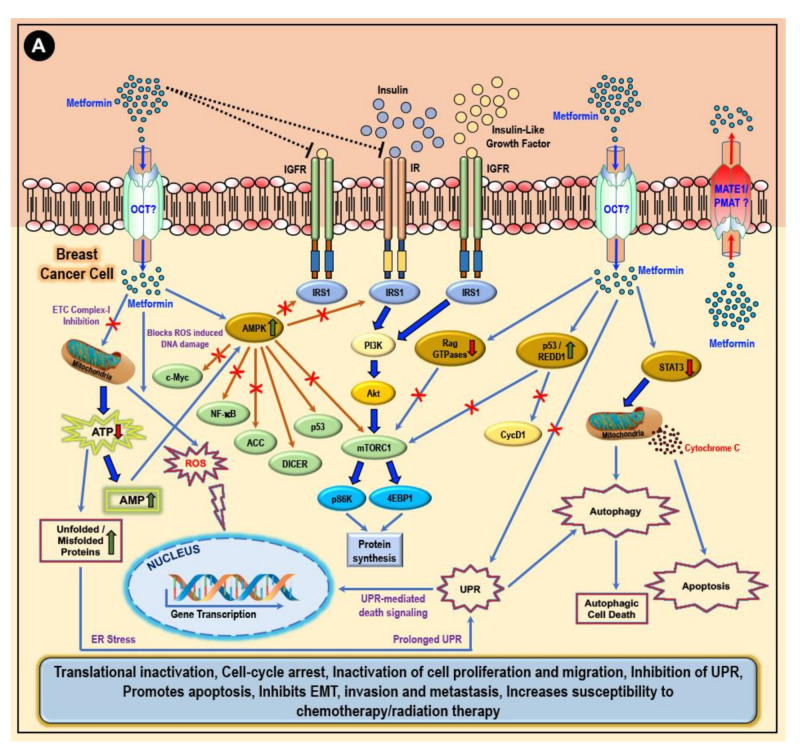

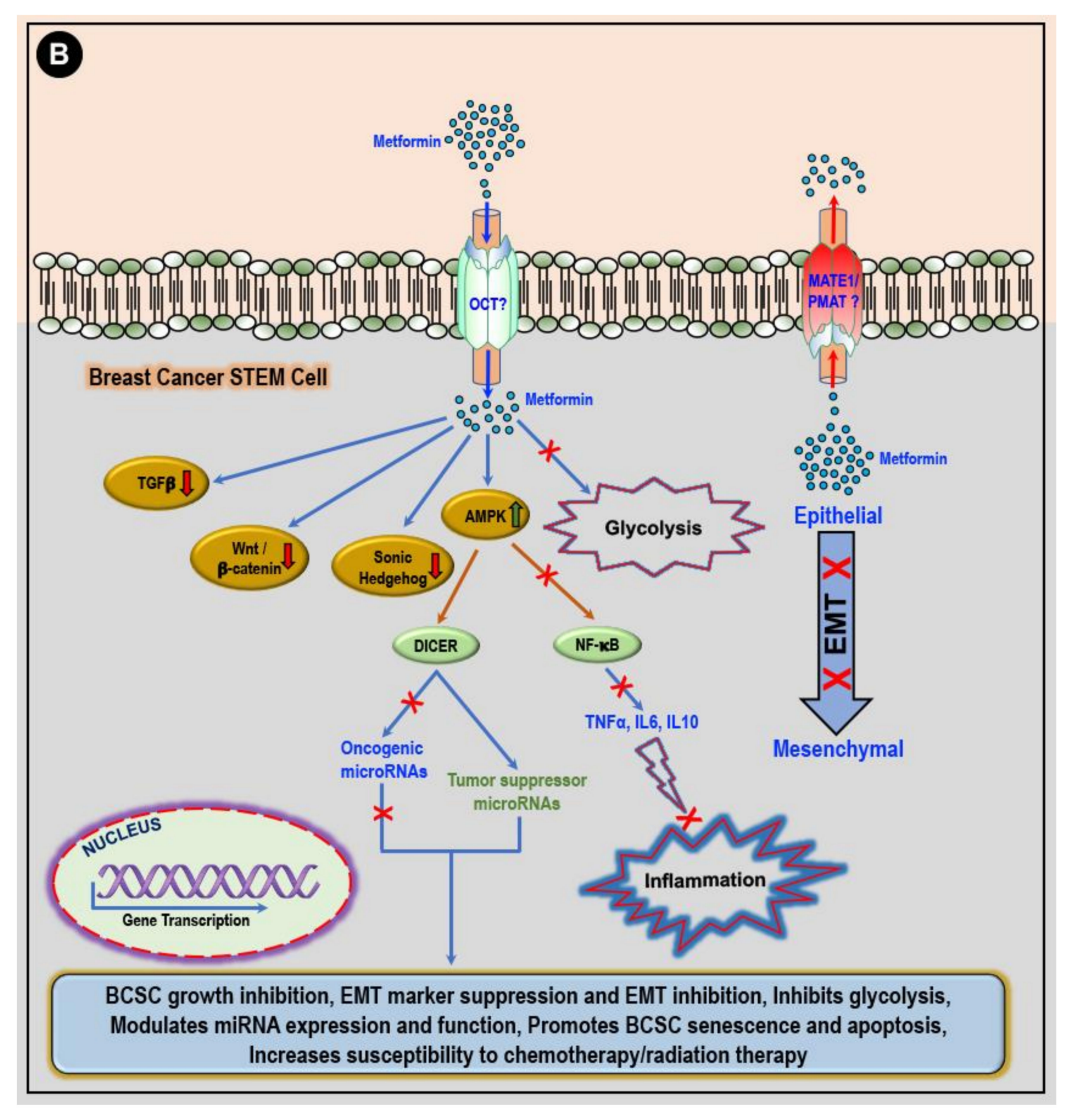
Known cellular anti-neoplastic and therapeutic resistance counteracting effects of metformin: (**A**) in breast cancer cells (figure adapted and modified from our published article Samuel, SM. et al. 2019 in Biomolecules [51] and (**B**) in breast cancer stem cells (BCSCs) (figure adapted and modified from Saini, N. et al. 2018 [202]. The hydrophilic and cationic metformin requires membrane-bound organic cation transporters (OCT) for intracellular transport and accumulation. In BCSCs, metformin treatment directly activates AMPK, and the ‘AMPK-dependent’ effects include NF-κB and DICER-mediated modulation of oncogenic and tumor-suppressor miRNA synthesis and expression. AMPK-independent metformin treatment-associated anti-cancer effects are mediated by the regulation of metabolism (inhibition of glycolysis) and transforming growth factor-beta (TGFβ), Wnt/β−catenin, and Sonic Hedgehog signaling mechanisms. Overall, metformin treatment in BCSCs causes BCSC growth inhibition, EMT marker suppression, and EMT inhibition, inhibits glycolysis, modulates miRNA expression and function, promotes BCSC senescence and apoptosis, and increases susceptibility to chemotherapeutic and radiotherapeutic intervention.

TNBCs have the highest number of CD44^+^/CD24^neg/low^ BCSCs when compared to the other molecular breast cancer subtypes [131,243]. Reports indicate that metformin is capable of selectively and preferentially targeting the BCSC side population of cells derived from breast cancer cells [243]. It impedes the BCSC cell proliferation and self-renewal, and it induces rapid cell death at physiological/much lower metformin concentrations than shown in most studies, which address the anti-neoplastic effect of metformin in the parental population of breast cancer cells [244,245,246]. Metformin exerts its anti-CSC effect by directly targeting genes related to BCSC growth and survival (such as Notch1, Oct4, Lin28, KLF4, Sox2, NF-κB, and MMP-2/9) and through various mechanisms that involve targeting (but are not limited to) (1) crucial genes and associated signaling pathways that contribute to BCSC, stemness, self-renewal, and metastasis, (2) inflammatory pathways, (3) metabolic pathways, and (4) miRNA-mediated pathways [192,202,243,247] (Figure 4).

Upregulation and increased levels of disheveled segment polarity protein 3 (DVL3) and β-catenin were found in the MCF7, MDA-MB-231, and T47D breast cancer cells when compared to healthy MCF10A breast cells [248]. The higher levels of DLV3 and β-catenin, key molecular players in the Wnt/β-catenin signaling pathway, enhanced breast cancer cell proliferation [248]. A dose-dependent depletion of DLV3, a subsequent decrease in β-catenin, and the inhibition of cell proliferation was observed in metformin (10 mM, 20 mM, and 40 mM)-treated MCF7 and MDA-MB-231 cells [248]. Compound C/dorsomorphin treatment suppressed the effect of metformin on disheveled 3 (DVL3) and β-catenin, indicating that the effect of metformin follows an AMPK-dependent mechanism [248]. However, this study used very high concentrations of metformin while still being unable to explain if these concentrations are achievable in a clinical setting. Furthermore, compound C was shown to inhibit several kinases other than AMPK non-specifically [84,249,250].

Fan et al. showed a metformin-related AMPK-dependent inhibition of Sonic Hh (SHh) signaling and the suppression of stemness and MSFE in CD44^+^/CD24^neg/low^ BCSCs derived from MDA-MB-231 cells [202,251]. In MDA-MB-231 breast cancer cells, metformin (1 mM, 3 mM, and 9 mM) treatment significantly decreased the mRNA expression and protein levels of SHh, Smo, Ptch, and Gli (components of the Hh signaling mechanism) and inhibited SHh-mediated Hh signaling-induced cell proliferation in a dose and time-dependent manner [251]. Similar results were obtained in MCF7 and BT549 cells [251]. Activation of the SHh signaling using a recombinant human SHh (rhSHh) subsequently increased the rate of cell proliferation and colony formation in the MCF7 and MDA-MB-231 cells. At the same time, metformin treatment attenuated the rhSHh cell proliferation and colony formation in these cells in a dose and time-dependent manner [251]. Similarly, the presence of metformin suppressed the rhSHh-accelerated growth of MDA-MB-231-inoculated tumor xenografts in Balb/c-nu mice [251]. Immunohistochemical analysis of the excised tumors revealed a significant decrease in Gli expression in the metformin-treated mice tumors compared to the higher expression of Gli in rhSHh-induced tumors [251]. Cell migration and invasions assays revealed a marked decrease in the migration and invasiveness of metformin-treated MDA-MB-231cells when compared to the increased rates of migration and invasiveness in rhSHh-activated cells [251]. Treatment with the SHh activator rhSHh increased the CD44^+^/CD24^neg/low^ sub-population of stem-cell-like cells and increased the stemness and MSFE of these cells derived from MDA-MB-231 cells [251]. On the other hand, metformin treatment caused a significant reduction in the CD44^+^/CD24^neg/low^ BCSCs in both non-activated and rhSHh-activated cells [251].

The induction of EMT, as well as its transdifferentiation and increased tumorigenic, migratory, and invasive capacity, was observed in TGFβ-treated transplantable mouse mammary epithelial CDβGeo cells when compared to non-treated cells [252]. Several studies reported a TGFβ-mediated EMT, self-renewal capacity, stemness, and resistance to therapy in breast cancers [141,253,254,255]. TGFβ was highly expressed in mesenchymal stem-like/claudin (MSL/CL) low breast cancer cells and was associated with a poor prognosis in patients with MSL/CL-like cancers [256]. The higher expression of TGFβ correlated to the activation of Sma-and mothers against decapentaplegic (SMAD) homolog protein and cell proliferation in mesenchymal (M; BT549) and MSL/CL (SUM159PT, MDA-MB-231, and Hs578T) cells [256]. Exposure to metformin (1.5 to 2.5 mM) alone and in combination TGFβ receptor I-kinase inhibitor (TβRI-KI) blocked the activation TGFβ signaling molecules Smad2, Smad3, Snail1 and inhibitor of differentiation 1 (ID1) in mesenchymal (BT549) and MSL/CL (MDA-MB-231 and MDA-MB-436) cells. At the same time, no effect was seen in TGFβRI-deficient MCF7 cells, which subsequently suppressed the TGFβ-induced motility and invasiveness of the cells [256]. A combination of metformin and shRNA-mediated TGFβ knockdown suppressed EMT and metastasis in CF41 cells (canine mammary metastatic cell line) tumor xenografts in athymic nude mice [257]. The presence of metformin decreased the levels of N-cadherin and vimentin while increasing the levels of E-cadherin and claudin-7 in both parental CF41 and TGFβ knockdown CF41 cells in vitro and in vivo [257]. In CD44^+^/CD24^neg/low^ and CD44^+^/CD24^+^ tumorigenic cell populations derived from basal-like breast cancer cells, non-cytotoxic concentrations of metformin (1 mM) suppressed the occurrence of stem-cell-like characteristics by the suppression of EMT [258]. Metformin treatment significantly downregulated the expression of EMT transcription factors ZEB1, TWIST1, and SNAI2 (Slug) and cytokines such as TGFβ that drive the process of EMT [258]. In turn, this effect reduced the MSFE and the ability of these metformin-treated CD44^+^/CD24^neg/low^ cells to form tumors [258]. However, a combination of aspirin and metformin reportedly increased the levels of TGFβ1 and tumor-suppressor properties of TGFβ1 in 4T1 mouse breast cancer cells and reduced cell viability and induced cell death in these cells [259]. Both tumor-promoting and tumor-suppressing effects of TGFβ with regard to cellular proliferation, invasion, and metastasis were reported [202].

The NF-κB pathway and its target genes that include cytokines (TNFα, IL1, IL6, and IL8) were crucial for cell survival and proliferation, anti-apoptosis, EMT, and metastasis in cancers [202]. In healthy MCF10A breast epithelial cells, the transient activation of Src triggered the transformation of these cells to gain stem cell-like features and increased the ability of the cells to form mammospheres [260]. Treatment with tamoxifen supported this phenotypic transformation and increased the cell motility and invasiveness and tumorigenic capacity of the cells in vivo in nude mice [260]. Further analysis revealed that NF-κB played an integral role in the transformation of these cells, the upregulation of CD44 expression, and the Lin28B-mediated downregulation of miRNA lethal-7a (let-7a) [260]. The treatment of cancer stem cell-like transformed MCF10A cells with metformin (100 μM) significantly inhibited the nuclear translocation of NF-κB and phosphorylation of STAT3 in the cancer stem cells, reduced the MSFE of these cells in vitro, and inhibited the tumorigenic capacity of these cells in vivo [245,261]. Besides, a combination of metformin and DOX suppressed tumor growth and prolonged the possible relapse of the tumor in vivo [261].

Metformin modulates various proteins involved in the miRNA biosynthesis pathway and miRNAs that regulate CSC genes related to stemness, self-renewal, and EMT [202]. In MCF7 cells treated with metformin (20 mM), the levels of miR-27a were significantly suppressed, while the levels of AMPKα2 increased and activated caspase-3-dependent apoptosis [262]. On the contrary, the overexpression of miR-27a (use of an miR-27a mimic) inhibited apoptosis, promoted cell survival and proliferation, and decreased the levels of AMPKα2, while an increase in the levels of AMPKα2 was seen in miR-27a-inhibited cells [262]. The upregulation of miR-34a and inhibition of the Sirt1/Pgc1α/Nrf2 pathway was implicated in the anti-proliferative effect of metformin in breast cancer cells [263].

EMT was enhanced in TGFβ−exposed MCF7 cells with a significant increase in miR-181a and enhanced the MSFE of these cells, in addition to the decrease in tumor suppressor miR-96 [264]. However, in the presence of metformin (1 mM, 10 mM), TGFβ failed to upregulate miR-181a and downregulate miR-96, which in turn also reduced the MSFE of the metformin-treated cells [264]. Metformin treatment also reportedly increased the expression of the miRNA let-7a in MCF7 cells [264].

In metformin (10 mM)-treated MDA-MB-468 TNBC cells, the expression of fatty acid synthase (FASN) was significantly decreased when compared to non-treated cells [265]. A similar decrease in FASN happened in metformin-treated BT549 and MDA-MB-231 cells [265]. Interestingly, while the decrease in FASN occurred when the cells were maintained at 5 mM glucose during metformin exposure, the effect of metformin treatment-associated decrease in FASN was absent in the presence of high glucose (17 mM) [265]. Micro-RNA expression profiling revealed the upregulation of miR-193a-3p and miR-193b in the metformin-treated TNBCs [265]. The metformin-induced miR-193b specifically targets FASN in breast cancer cells while having little effect on healthy cells [265]. The ability of metformin to decrease FASN, reduce MSFE in CD44^+^/CD24^neg/low^ and ALDH^+^ cells, and initiate apoptosis was markedly compromised upon the inhibition of miR-193b [265].

Metformin treatment (1 mM and 5 mM) reduced the levels of Rab27A, a member of the RAS oncogene family, in a dose-dependent manner [266]. In addition, the combination of downregulation of Rab27A and metformin treatment was more effective in significantly decreasing the MDA-MB-231 TNBC-derived CD44^+^/CD24^neg/low^ cell population and inhibition of MSFE when compared to either Rab27A downregulation or metformin treatment alone [266].

In an ER–Src-inducible model of transformed (tamoxifen-induced) MCF10A cells, the cellular transformation was associated with metabolic alterations commonly associated with cancer cells, such as higher rates of glucose and glutamine uptake and subsequent lactate and ammonium production [267]. While treatment with metformin (300 μM) was associated with an increase in glucose uptake and lactate production, interestingly, however, metformin prevented the transformation-associated increase in glycolytic intermediates (fructose 1, 6, bisphosphate, dihydroxyacetone phosphate, and glyceraldeyde-3-phosphate) [267]. Furthermore, metformin treatment increased the levels of glycerol-3-phosphate in the transformed cells [267]. A metformin treatment-associated decrease in the levels of Kreb’s cycle intermediates was also observed in the transformed and CAMA-1 cells (a stably transformed breast cancer cell line) [267]. Interestingly, metformin treatment selectively and preferentially depleted the nucleotide triphosphates in the BCSCs of the mammospheres derived from the CAMA-1 cells, while this effect was not seen in parental CAMA-1 cells [267]. Similar effects were observed with the more potent biguanide, phenformin [267]. In MCF7-derived CD44^+^/CD24^neg/low^ BCSCs (which showed a significantly lower ROS level when compared to the parental population of the cells), treatment with metformin (5 mM) markedly increase the ROS levels in these cells. The increased ROS levels and the resultant oxidative stress are correlated to increased DNA damage-induced apoptosis and a reduction in cell survival in cancers [202,268,269].

### 5.2. Metformin and Counteracting Therapeutic Resistance in BCSCs

Tamoxifen treatment of human mammary epithelial cells (MCF-10A) that express ER-Src (a fusion of the v-Src oncoprotein with the ligand-binding domain for ER) induced the transformation of at least 10% of these cells to CD44-expressing CSCs with an ability to form mammospheres in culture [245]. Metformin treatment rigorously inhibited the transformation of the MCF-10A cells during tamoxifen exposure, reduced invasive growth during wound-healing experiments, and suppressed colony formation and the generation of mammospheres [245]. Additionally, metformin pre-treated MCF-10A cells failed to generate tumors in vivo, while non-treated transformed MCF-10A cells formed tumors when injected into nude mice [245]. Furthermore, MCF7, SKBR3, and MDA-MB-468 cells that contain a minority population of CSCs showed a reduction in the number of mammospheres following metformin treatment [245]. Interestingly, metformin (0.1 mM to 0.3 mM) had a preference for targeting the sub-population of CD44^+^/CD24^neg/low^ cancer stem cells within a population of the transformed MCF-10A and MCF7 cells [245]. In fact, this landmark study demonstrated for the first time the ability of metformin to target CSCs that are resistant to standard chemotherapeutic approaches [245]. In type 2 diabetic subjects on an oral metformin treatment regimen, the peak metformin concentrations in the plasma and/or hepatic circulation ranges between 20 and 100 μM [3,51,52,250,270,271]. Interestingly, in the study by Hirsch et al. (2009), metformin at concentrations of 0.1 mM to 0.3 mM (although still higher by nearly 10-fold when compared to the hepatic/plasma concentrations achieved by a type 2 diabetic subject on oral metformin treatment regimen) was effective against the self-renewing tumorigenic CSCs, while it did not have any significant effect on the non-stem cell population [245]. In the in vivo tumor model generated by injecting transformed MCF-10A cells into nude mice, the administration of metformin and DOX synergistically eliminated the tumor, while DOX alone was found to be less effective, and metformin alone failed to show any response [245].

In HER2 overexpressing (tumor-initiating breast cancer stem cell-like), SKBR3 cells with an ‘acquired resistance’ to trastuzumab and JIMT-1 cells (HER2 overexpressing cells derived from the pleural metastasis of a patient who was clinically resistant to trastuzumab ab initio) with an ‘intrinsic’ refractoriness to trastuzumab, treatment with metformin (0.05 mM, 0.1 mM, 0.5 mM, and 1 mM) inhibited the MSFE and hence reduced the number and size of mammospheres in a dose-dependent manner [113,246]. The synergistic action of metformin and trastuzumab downregulated the percentage of trastuzumab-resistant CD44^+^/CD24^neg/low^ JIMT-1 cells with stem-cell-like capabilities, such as self-renewal, which act as progenitor cells for the formation of new breast neoplasms [113,246]. In another study, while an extended 7-week treatment with trastuzumab failed to inhibit the growth and reduce the size in JIMT-1 tumor xenografts, the systemic administration of metformin (as a monotherapy) correlated with a significant 2-fold reduction in tumor volume [113,244]. However, a combination of metformin and trastuzumab was effective in further reducing the tumor volume (by 4-fold) when compared to using metformin as a single treatment agent [113,244]. Additionally, it was observed that metformin administration specifically and preferentially caused cell death in CD44^+^/CD24^neg/low^ (>10-fold, IC_50_ of metformin = 1 ± 0.2 mM) breast CSCs when compared to the effect of metformin (IC_50_ of metformin = 11 ± 2 mM on non-CD44^+^/CD24^neg/low^ immunophenotypes of the cells [113,244,246]. The preferential cytotoxicity of metformin (0.5 mM, 1 mM and 5 mM) toward CD44^+^/CD24^neg/low^ MCF7-derived BCSCs was also reported by Lee et al. in 2014 [272]. Here, they showed that a combination of metformin (5 mM) treatment and hyperthermia (incubation at 42 °C for 1 h) was more efficient in causing BCSC death when compared to hyperthermia or metformin treatment alone [272].

Mammosphere-associated CD44^+^/CD24^neg/low^ MDA-MB-468-derived breast CSCs possessed a 10-fold higher resistance to DOX, PTX, and FEC when compared to parental MDA-MB-468 cells [127]. On the other hand, the CD44^+^/CD24^neg/low^ MDA-MB-468-derived breast CSCs showed significant sensitivity and susceptibility to metformin while they remained resistant to other known AMPK activators such as AICAR and A-769662 [127]. The synergistic sensitization to FEC, enhancement of anti-proliferative effect, and induction of apoptosis was observed in metformin-treated (2.5 mM to 20 mM) CD44^+^/CD24^neg/low^ MDA-MB-468, MCF-7, and SKBR3-derived breast CSCs [127]. The depletion of endogenous AMPK using siRNA did not have any significant effect on the effect of metformin on the breast CSCs, indicating the involvement of an AMPK-dependent mechanism [127]. The combination of metformin and FEC exhibited a significant synergistic effect in both parental and CD44^+^/CD24^neg/low^ breast CSCs, while the combination of metformin and DOX was effective only in CD44^+^/CD24^neg/low^ breast CSCs. Furthermore, in the CD44^+^/CD24^neg/low^ breast CSCs, the presence of metformin increased the cellular uptake of glucose and subsequent production and accumulation of lactate in the cells; however, the depletion of cellular ATP levels in the metformin-treated breast CSCs severely impairs the DNA repair mechanisms that are required by the CSCs to repair the FEC-induced DNA damage [127].

Metformin treatment markedly decreased the percentage of the tumor-forming TNBC stem cell population in HCC1806 and HCC1937 cells and reduced the MSFE of the treated cells when compared to the non-treated cells [273]. Furthermore, in an in vivo mice model, it was observed that the tumor-forming efficiency of the HCC1806 cells significantly decreased upon metformin pre-treatment, while it also extended the tumor-free survival of the mice and decreased the weight and volume of the induced tumor when compared to mice in which the non-treated cells were seeded [273]. The metformin treatment-associated protein kinase-A (PKA) and glycogen synthase kinase-3β (GSK-3β)-dependent downregulation of the expression of the stem-cell transcription factor Krüppel-like factor 5 (KLF5) and its downstream targets (Nanog and FGF-BP1) were in part responsible for the effect of metformin on the TNBC cells [273]. However, the major drawback of the study lies in the fact that very high concentrations of metformin (20 mM and 50 mM) were used to arrive at the conclusions mentioned in the study [273].

Bone metastases, in patients with advanced breast cancer, are often treated with denosumab (trade name: Prolia^®^ and Xgeva^®^), which is a human monoclonal antibody against receptor activator of nuclear factor-kappa B ligand (RANK-L) [274]. However, in BRCA1 mutation carriers, BRCA1 haploinsufficiency-driven overexpression of RANK-L was observed, which correlated to the RANKL-addicted cancer stem cell-like behavior within cell populations with the BRCA1 mutation [274]. BRCA1-mutated basal-like breast cancer cells exhibited primary resistance to denosumab in mammosphere assays, thereby leading to an increased propensity in developing aggressive breast neoplasms and increasing the bone metastasis-initiation capacity of these cells [274]. However, metformin (5 mM) treatment suppressed the BRCA1 haploinsufficiency-driven overexpression of RANK-L and synergistically reduced the self-renewal capacity in BRCA1-mutated basal-like breast cancer cells, resulting in a decrease in the breast cancer-initiating cell population [274].

Low levels of miR-708 were observed in breast cancer specimens when compared to the healthy tissue, and decreased levels of miR-708 reportedly corresponded to increased chemoresistance in patients who do not respond favorably to neoadjuvant chemotherapeutic intervention [275]. MCF7 and MDA-MB-231-derived BCSCs were highly capable of self-renewal and tumorigenesis, as evidenced by the increased MSFE in vitro and the tumor initiation potential in vivo (in BALB/c mice) [275]. The miR-708 significantly decreased in these mammospheres and tumors [275]. Furthermore, miR-708 was significantly downregulated in the stem-cell-like CD44^+^/CD24^neg^ population of the MCF7 and MDA-MB-231 cells that were capable of self-renewal and tumorigenesis when compared to non-stem cell population. At the same time, the overexpression of miR-708 markedly reduced the stem-cell-like CD44^+^/CD24^neg^ population and the stemness and tumorigenic capacity of these cells in vivo [275]. Knocking down miR-708 in adherent parental MCF7 and MDA-MB-231 cells drastically enhanced the MSFE of these cells [275]. The miR-708 was significantly downregulated in MCF7 cells with acquired resistance to adriamycin (MCF7/ADR) when compared to parental MCF7 cells [275]. In fact, MiR-708 inhibited the stemness of derived BSCSs via its inhibitory effect on the expression of CD47, which was further confirmed by the fact that knocking down CD47 by using shCD47 decreased the stem-cell-like CD44^+^/CD24^neg^ population of the cells [275]. Either the overexpression of miR-708 or knocking down CD47 expression sensitized MDA-MB-231 cells to docetaxel [275]. The treatment of BCSCs derived from MCF7 and MDA-MB-231 with metformin (10 mM) significantly increased the expression of miR-708, which in turn inhibited the CD47 mRNA expression [275]. Consequently, a dose-dependent decrease in the protein levels of CD47 was observed in cells treated with metformin (0.3 mM, 1.0 mM, and 3.0 mM) [275].

A combinatory therapeutic approach using metformin (1 mM or 5 mM) and radiation (4 Gy) significantly increased the levels of phospho-AMPK and phospho-ACC and decreased phospho-mTOR (and its downstream targets phospho-S6K1 and phospho-4EBP1) compared with metformin or radiation alone [276]. Suppression of the AMPK levels by siRNA attenuated the ability of metformin to sensitize the cancer cells to radiation therapy and cause cell death, which is indicative of an AMPK-dependent mechanism for the effect of metformin [276]. The percentage of the cancer stem-cell-like CD44^+^/CD24^neg/low^ sub-population of MCF7 cells increased upon irradiation and subsequent incubation and were reportedly resistant to radiation (3–5 Gy) than the non-CSC parental population of MCF7 cells [276]. Interestingly, the post-irradiation (3–7 Gy) incubation of the cells in metformin (1 mM) for 48 h suppressed the irradiation-dependent increase in the tumorigenic stem-cell-like CD44^+^/CD24^neg/low^ fraction of MCF7 cells [276]. Similar results were obtained when these experiments were conducted in FSaII mouse fibrosarcoma cells, since the post-irradiation incubation of the cells in metformin (1 mM) for 48 h suppressed the irradiation (3–5 Gy)-dependent increase in the ALDH1-positive fraction of FSaII cells [276].

### 5.3. Advantages of Combinatory Metformin and BCSC Targeted Therapy over Conventional Cancer Cell-Targeted Therapy

Metformin can significantly reduce cancer risk and the progression and aggressiveness of cancer through various proven molecular mechanisms [51]. The multifaceted anti-neoplastic potential of metformin has rigorously thrust this widely prescribed anti-hyperglycemic drug into the limelight as a candidate drug with significant potential to be ‘re-purposed’ for the treatment of several different cancers. Furthermore, metformin (1) use is not regulated by any patent regulations, (2) is safe and well-tolerated, with minimal/rare side effects, and (3) can be easily synthesized, is economical, and is marketed globally [51].

**Figure 5:**
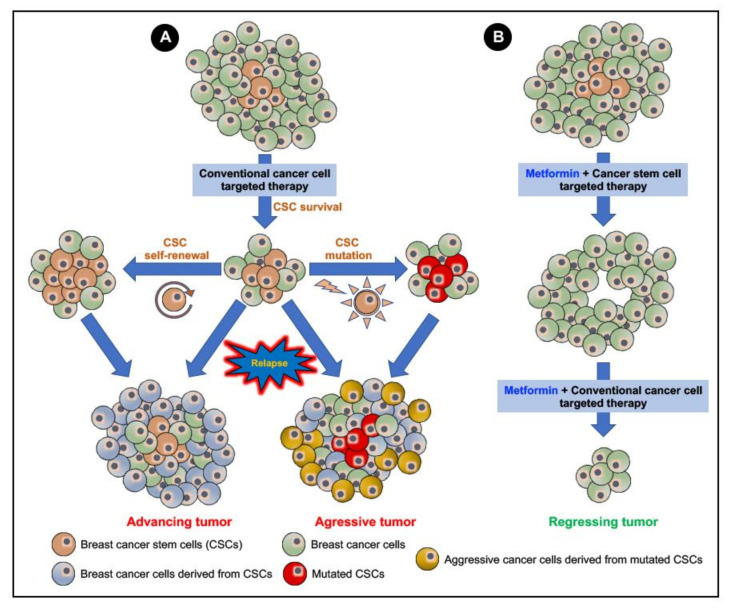
Efficacy of a combinatory metformin and breast cancer stem cell (BCSC) targeted therapy over conventional cancer cell-targeted therapy: A conventional anti-cancer therapy targeting terminally differentiated cancer cells (**A**) should efficiently inhibit cell proliferation and induce the apoptotic cell death of the cancer cells. However, the less abundant CSCs effectively evade therapeutic intervention. Then, the CSC through a process of self-renewal and generation of tumor progenitor cells and mutations mainly causes a relapse/recurrence of more aggressive, invasive, and metastatic forms of the neoplasm. Using metformin in combination with a cancer stem cell-targeted therapy (**B**) will effectively target and kill the CSCs, which then can be followed up with a combinatory treatment using metformin and the conventional anti-cancer therapy to cause tumor regression, effectively cure cancer, and avoid cancer relapse/recurrence.

In 2009, Hirsch et al. first reported that metformin preferentially inhibited BCSCs in preclinical models of breast cancer [245,277]. Similar results were also later observed later in prostate and lung cancer cell lines [277,278]. Since then, metformin was shown to be effective in selectively and effectively targeting CSCs in many different cancers by (1) targeting the altered metabolism, (2) inhibiting EMT, (3) sensitizing the CSCs to anti-cancer agents, (4) targeting specific miRNAs, (5) modulating multi-drug transporter proteins, (6) suppressing several pro-oncogenic/anti-apoptotic genes and signaling pathways, and (7) activating various anti-oncogenic/pro-apoptotic genes and signaling pathways [202,279,280]. The available data point toward the following advantages of targeting BCSCs using metformin over conventional cancer cell-targeted therapy (Figure 5):

(1) The BCSCs, while resistant to several routinely used anti-cancer agents, are sensitive to metformin. The less abundant CSCs that survive the administration of the routinely used anti-cancer agents are ultimately responsible for differentiation into tumor progenitor cells that cause a relapse/recurrence of cancer. Besides, CSCs can self-renew and may also undergo mutation to give rise to advanced and aggressive forms of the tumor [127,244,245,246,275,276,281].
(2) Metformin selectively and preferentially targets BCSCs at lower concentrations, while a high concentration of metformin is required to effectively target terminally differentiated cancer cells [88,93].
(3) Metformin treatment re-sensitizes the CSCs to multiple drugs and radiation, thus making the CSCs more susceptible to the routinely used standard anti-cancer agents and therefore improving the efficacy of the therapeutic intervention [127].
(4) Combinatory metformin therapy to target CSCs increases the efficacy of the treatment and improves the overall outcome and prognosis. The inhibition of invasiveness and metastasis dramatically reduces the chances of cancer relapse/recurrence. Furthermore, the development of acquired resistance to drugs can be minimized when used in combinations [127,244,245,246,275,276,282].

## 6. Challenges and Future Directions

### 6.1. Intrinsic and Acquired Resistance to Metformin

Several studies have shown the metformin treatment-related (1) sensitization of cancer cells and CSCs and (2) re-sensitization of resistant cancer cells and CSCs to anti-cancer/cytotoxic drugs and radiation therapy. The possibility that certain cancers and CSCs possess ‘intrinsic’ resistance mechanisms and also develop ‘acquired’ resistance against metformin cannot be written off. There are only a handful of studies that have reported the resistance to metformin in breast cancers [283,284,285].

The acquired resistance to metformin was found to reprogram the transcriptomic signature in MCF7 cells toward the emergence of individual cellular states of the breast neoplasm supported by the activation and expression of genes that support stemness and metastasis [284]. The reprogrammed transcriptomic signature included the activation and expression of genes related to cancer cell migration and invasion, stem cell markers, and lipases that support metastasis [284]. However, the study used supra-physiological concentrations of metformin (5–50 mM). Scherbakov et al. (2016) reported that in sensitive MCF7 cells, treatment with metformin (10 mM) was accompanied by AMPK activation, and the inhibition of the NF-κB pathway, cyclin D1, and ERα, thereby suppressing cell proliferation [285]. On the other hand, the MCF7 breast cancer cells developed an Akt/Snail1/E-cadherin signaling axis-mediated dual resistance to tamoxifen and metformin when subjected to long-term exposure to metformin alone [285]. In another study, although the reported IC_50_ for adherent monolayers of MDA-MB-231 and MDA-MB-436 claudin-low subtypes of TNBCs were 33.0–51.8 mM and 25.1–30.7 mM, respectively, metformin at 2 mM concentration was sufficient to suppress the MSFE by 30.2% in MDA-MB-231 and 28.4% in MDA-MB-436 cells, indicating that sub-populations of CSCs derived from the MDA-MB-231 and MDA-MB-436 cells were susceptible to metformin at lower concentrations [283]. However, it must be noted that long-term exposure (>8 weeks) of metformin (2 mM) initiated a metabolic adaptation that increased the rate of glucose uptake and glycolysis in the TNBCs, which in turn compensated for the inhibition of the mitochondrial oxidative phosphorylation and electron transport chain [283]. These metabolic adaptations conferred resistance to metformin and promoted the accumulation of TNBC-derived BCSCs, which could ultimately lead to cancer cell invasion, metastasis, and a relapse of cancer [283]. However, the dependency of these cells on glycolysis increased the susceptibility of the sub-population of tumorigenic cells to therapeutic strategies that target glycolysis and/or glucose metabolism [283].

It must be taken into account that a majority of studies involving cancer cells, including breast cancers, are performed under high-glucose (25 mM) culture conditions, hence requiring very high concentrations of metformin to exert its cytotoxic and anti-neoplastic effect [286]. TNBCs maintained under high glucose conditions were typically resistant to metformin treatment owing to the altered glucose metabolism and hyperactivated glycolytic pathway that provided the necessary energy to drive the cellular processes that lead to cell proliferation and survival [287]. The high-glucose (25 mM) culture conditions mimic a diabetic condition. Although useful in explaining the possible anti-cancer/anti-tumor potential of metformin in diabetic subjects, studies conducted under glucose conditions ranging from zero glucose to hypoglycemia, normoglycemia, and hyperglycemia will be necessary to explain the anti-neoplastic effect and the variations thereof of metformin on different populations/sub-populations of cancer cells and CSCs. Furthermore, co-morbidities that pose a plausible risk factor for cancer incidence and progression must be considered while investigating potential drugs, such as metformin, in the view of the metabolic, epigenetic, and genetic alterations that present along with co-morbidities and can alter the course of cancer and the response to therapeutic intervention.

### 6.2. Targeted Drug (Metformin) Delivery Systems for Improved Therapeutic Efficacy

The in vivo and clinical data on the anti-neoplastic ability of metformin are still lacking and a little less convincing than the positive data available from several different in vitro studies conducted in various cancers. The results obtained from the in vitro experiments do not translate as effectively in the in vivo and/or clinical setting owing to the short half-life (5 h) of metformin and its compromised bioavailability, thus hindering its effective and full-fledged application as an anti-cancer drug [288,289,290]. In such a scenario, higher/repeated doses for longer durations may be required to initiate and sustain a positive remedial response in cancers [290,291,292]. As mentioned earlier in this article, owing to the hydrophilic and cationic nature of metformin at physiological pH, the efficiency of metformin depends on the membrane-bound transporter molecules for its cellular absorption (OCT1, OCT2, OCT3), distribution within the cells, and elimination (MATE1 and PMAT) from cells [60,61,62,63,64,65,66,67]. Metformin will be useful in suppressing cell proliferation and inducing apoptosis in cancer cells and CSCs that express OCTs, which support the intracellular accumulation of metformin, while, cancer cells, and CSCs that overexpress metformin extrusion transporters (MATE1 and PMAT) will confer resistance to metformin treatment [71,72,73,74].

Issues related to reduced drug absorption, rapid detoxification, and excretion of the drug—thus affecting the overall drug availability and the requirement of sub-optimal doses in patients who present with lower tolerance to the drug—calls for precise and targeted drug delivery systems [21]. Nanoparticle-based drug delivery are currently being tested in clinical trials and represents a way of focalized drug delivery to tumors, thereby reducing toxicity and side effects while increasing the bioavailability and anti-tumor efficacy of the drugs [293,294,295]. In this regard, novel metformin formulations that enhance its bioavailability and decrease its dosing frequency and duration are being studied [291]. A better option should be to attain a localized delivery of metformin to the affected tissue/cells. Several studies have successfully reported the efficacy of metformin administration using micro/nanoparticle drug delivery systems. MiaPaCa-2 pancreatic cancer cells were more susceptible to the cytotoxic effects of metformin-encapsulated *O*-carboxymethyl chitosan (*O*-CMC) nanoparticles (240 ± 50 nm) when compared to healthy cells [288]. Although both the MiaPaCa-2 cancer cells and healthy cells non-specifically internalized the metformin-loaded nanoparticles, the metformin released from the nanoparticles was preferentially cytotoxic toward the MiaPaCa-2 cancer cells [288]. Moreover, poly (lactic acid-co-glycolic acid)-polyethylene glycol (PLGA–PEG) nanoparticles loaded with metformin displayed significantly higher cytotoxicity in MDA-MB-231 and T47D cells than free metformin [291]. The triple-negative MDA-MB-231 cells were more susceptible (lower IC_50_) to the PLGA–PEG–metformin nanoparticle than the ER^+^/PR^+^ T47D cells [291]. In T47D cells, treatment with PLGA–PEG co-encapsulated metformin and curcumin in combination showed a dose-dependent cytotoxic effect with higher efficiency than free forms of metformin or curcumin alone and a combination of free forms or metformin and curcumin [296]. The PLGA–PEG–metformin nanoparticle and the PLGA–PEG co-encapsulated metformin and curcumin inhibited the expression of human telomerase reverse transcriptase (hTERT), which is a component of the telomerase enzyme complex, with a higher efficiency than free metformin in the breast cancer cells [291,296]. The increased activity of telomerase was implicated in almost all cases of breast cancers (approximately 90%) and breast cancer cells, which help the cells bypass normal cellular senescence and bestow them with rapid proliferation potential [291]. The metformin-induced therapeutic re-activation of telomere shortening by the inhibition of telomerase activity in cancer cells is an exciting approach to curb the proliferation of cancer cells and suppress the growth of the tumor [51,291]. In addition, metformin delivery using a liposome-based nanoparticle delivery system was superior in its efficacy (lower IC_50_) in MDA-MB-231 and MCF7 cells when compared to free metformin. The positively charged metformin-loaded liposomes inhibited the cell migration and colony formation potential of the breast cancer cells while activating apoptotic cell death following the treatment [297]. The anti-tumor activity of the metformin-loaded liposomes was confirmed in a clinically relevant tumor simulation model in vitro [297].

Baldassari et al. (2018) demonstrated the anti-tumor efficacy of the focalized delivery of metformin to the proximity of the tumor using an injectable slow/delayed-release formulation of metformin [292]. The metformin formulation (stable at 5 °C for one month), a solution at room temperature, once injected into the body, is converted to the gel in response to the higher body temperatures [292]. Multiple administrations (100 mg) of the injectable formulation of metformin to the proximity of the tumor site in MDA-MB-231/luc^+^ breast cancer cell xenografts in NOD/SCID mice caused an accumulation of metformin in the tumor tissue at significantly higher concentrations than the plasma concentration achieved during oral metformin administration [292]. This accumulation of metformin correlated to the inhibition of cellular proliferation, reduction in tumor growth, increase in caspase-3 dependent apoptosis, and decrease in the levels of phospho-ERK1/2 [292]. More studies are warranted to study the efficacy of metformin delivery by advanced drug delivery systems to delineate its anti-neoplastic effect, and the variations thereof, in different populations/sub-populations of cancer cells and CSCs.

### 6.3. Lack of Data from Clinical Trials

Data from ClincalTrials.gov enlisted 353 clinical trials at various stages (search keywords: cancer + metformin, search performed on 29 June 2020; Table 2) that involve the testing of metformin (a majority of the studies involve metformin in combination with other anti-cancer drugs/agents) in cancers. Among the total enlisted 353 clinical trials, only 41 trials have reported their results.

At least 48 out of the 353 clinical trials are breast cancer-specific and involve the testing of metformin specifically in different breast cancers (search keywords: breast cancer + metformin, search performed on 29 June 2020; Table 2). In a majority of the breast cancer clinical trials, metformin is used in combination with one/more anti-cancer agents or radiation (Table 3). Twenty of the breast cancer and metformin-related clinical trials are active, while 17 were completed, and 11 were either terminated, withdrawn, or the status remains unknown (Table 2). Interestingly, excluding the 20 currently active clinical trials (at various stages of recruitment of test subjects), out of the 23 (17 completed and 6 terminated) metformin and breast cancer-specific clinical trials, the data have been reported by only 6 of those clinical trials (Table 2).

As noted in our previous article, there seems to be an under-reporting of data obtained from a majority of the clinical trials in which metformin was tested as the sole or one of the interventions during the trial [51]. While clearly understanding the impact and importance of the several cell-based and in vivo studies that substantiate the anti-tumor potential of metformin, it is equally, if not more crucial, to publish/reveal the outcomes of ongoing clinical trials that aim at ‘re-purposing’ metformin as an anti-cancer drug.

### 6.4. Future Directions

Metformin has emerged as a prime candidate drug that is cost-effective and has minimal side effects, which can be essentially ‘re-purposed’ as an effective anti-neoplastic agent. In this regard, the global attention that metformin has gained over the years is based on the abundantly available epidemiologic, meta-analysis, and preclinical data.

Among the several ongoing clinical trials, there are very few that intend to study the effect of metformin as a monotherapy or in combination with other anti-cancer drugs specifically on CSCs (Table 4). The data currently available on metformin’s ability to selectively/preferentially target CSCs is overshadowed by the abundance of data available on the anti-neoplastic potential of metformin on parental/terminally differentiated cancer cells. However, more recently, metformin has emerged as crucial anti-CSC targeting drug that can efficiently combat the prime problems of drug resistance and cancer relapse, which always has troubled cancer biologists and researchers. Several different signaling pathways, such as the AMPK/mTOR/PI3K, insulin/IGF1, Ras/Raf/Erk, SHh, Wnt, TGFβ, Notch, and NF-κB signaling pathways have been implicated in the ability of metformin to selectively inhibit CSCs and thereby suppress cell proliferation, self-renewal, differentiation, metastasis, and metabolism. There is no doubt that the preclinical data available are promising; however, the challenge remains as to how these preclinical data translate in a clinical scenario. Previously, we have noted that in a vast majority of the cancer-related clinical trials, metformin is most frequently studied as a co-treatment with other routinely used chemotherapeutic drugs and rarely as a monotherapeutic agent, and hence, it is unlikely that metformin will gain importance as a monotherapy in cancer treatment [51,298]. Hence, more clinical trials are warranted to identify and verify the immediate targets, crucial mediators, and regulatory components that can explain the demonstrated (in several in vitro and in vivo studies) selective anti-CSC effects of metformin when used as a sole agent or in combination with other anti-cancer drugs. It remains imperative to assess whether patients with metastatic, aggressive, and drug-resistant/refractory forms of cancers respond to metformin. A better understanding of the effect of anti-cancer metformin in the clinical realms of the disease will help oncologists optimize therapeutic interventions in their treatment subjects. At some point along the way, this will also help researchers answer the lingering question as to whether metformin, an antidiabetic drug with anti-cancer potential, is safe in non-diabetic cancer patients and whether diabetic and non-diabetic individuals who are at high risk (for various reasons) of developing cancers would benefit from taking metformin as a cancer-preventive drug.

## 7. Conclusions

The occurrence of intrinsic and acquired therapeutic resistance remains a major hurdle faced by the clinician during the course of the treatment of cancer. From a cancer patient’s point of view, apart from the debilitating side-effects that one suffers during the course of the treatment, there is nothing more depressing than the fear of relapse/recurrence of the disease due to the ineffectiveness of the treatment. Therefore, counteracting therapeutic resistance remains a key challenge that determines the efficacy of cancer treatment and the overall outcome and impact of the disease in the lives of affected individuals.

In this review, we have detailed how breast cancer stem cells (BCSCs) contribute to drug/therapeutic resistance in breast cancers and discussed how targeting the various aspects of BCSC conferred drug/therapeutic resistance could in turn sensitize breast cancers to therapeutic intervention and prevent relapse/recurrence of the disease. Furthermore, the current data available on the anti-neoplastic effects of metformin (the most widely prescribed anti-diabetic drug) makes it an interesting candidate for drug re-purposing for the treatment of cancers. In this regard, we have examined and discussed the available data (in vitro, in vivo and clinical data) on how targeting BCSCs using metformin can counteract BCSC-related therapeutic resistance, which when followed by conventional anti-cancer therapy could prove to be more efficient in the treatment of breast cancers. However, the possibility of the development of an ‘acquired’ resistance to metformin cannot be ignored and must be subjected to detailed studies. While majority of the available data on the efficacy of metformin in targeting BCSCs is linked to in vitro and in vivo experiments the major setback is the lack of translational clinical trials and data that addresses the challenges faced in an actual clinical setting. More clinical studies are warranted to address the efficacy metformin in targeting BCSCs and to test the efficacy of targeted drug delivery systems for an improved therapeutic outcome. Additionally, the inter-tumor and intra-tumor heterogeneity and plasticity in cancers, including breast cancers, calls for specific focus on the various aspects of predictive, preventive and personalized medicine (PPPM/3PM) for a better treatment outcome on a case-by-case basis.

## Figures and Tables

**Table 1:** Commonly used chemotherapeutic drugs in breast cancer and pathways or factors involved in drug resistance.

Cell Lines	Drug Intervention	BCSC Marker Status		Pathways/Factors Involved in Drug Resistance	Reference
		CD44^+^/CD24^neg/low^	ALDH^+^		
MCF7	PTX, DOX, Epi, 5-FU, FAC, Mito	↑	---	Notch, NRF2, NF-κB/IL6	[192,203,204,205,206,207]
	PTX, DOX	---	↑	ALDH, PHGDH	[192,208,209]
T47D	DOX, Cis, 5-FU, FAC	↑	---	Hh, MCL1, XIAP, NF-κB/IL6	[192,206,210,211,212]
BT474	FAC, Trastuzumab	↑	---	NF-κB/IL6, IL6R	[192,206,213]
SUM190	PTX, DOX, 5-FU	↑	---	NF-κB/STAT3	[192,205]
SKBR3	DTX, 5-FU	↑	---	Hh, XIAP	[192,210,211]
MDA-MB-231	DOX, Epi, Noco, FAC	↑	---	GRP78, NF-κB/IL6	[192,203,206,214]
	PTX, DTX, DOX	---	↑	FAK, autophagy, DNMT, PHGDH, HIF1α, HIF2 α	[156,192,215,216,217,218,219]
SUM159		---	↑	ALDH, PI3K/Akt/mTOR, NF-κB, Hh, CXCR1, HIF1α	[192,219,220,221,222]

5-FU = 5-fluorouracil, ALDH = aldehyde dehydrogenase, Cis = cisplatin, CXCR1 = (C–X–C motif) receptor 1, DNMT = DNA-methyl transferase, DOX = doxorubicin, DTX = docetaxel, Epi = epirubicin, FAC = 5-FU+DOX+cyclophosphamide, FAK = focal adhesion kinase, GRP78 = glucose regulated protein 78, Hh = Hedgehog, HIF1α/2α = hypoxia-inducible factor 1/2 alpha, IL6 = interleukin 6, IL6R = IL6 receptor, MCL1 = myeloid cell leukemia 1, Mito = mitomycin, mTOR = mammalian target of rapamycin, NF-κB = nuclear factor kappa-B, Noco = nocodazole, NRF2 = nuclear erythroid-related factor 2, PHGDH = phosphoglycerate dehydrogenase, PTX = paclitaxel, PI3K = phosphatidylinositol-3-kinase, STAT3 = signal transducer and activator of transcription 3, XIAP = X-linked inhibitor of apoptosis. This table was adapted from Dittmer, J. (2018) [192].

**Table 2:** Status of metformin administration-related clinical trials in various cancers (https://clinicaltrials.gov/).

Serial No:	Type of Cancer	Total Number of Registered Trials	Completed	Active, Not Recruiting	Active, Recruiting/Not Yet Recruiting	Terminated	Withdrawn/Suspended	Unknown Status#	Has Results *
1	All Cancers	353	142	29	79/14	34	12 / 7	38	41
2	Lung Cancer	20	5	1	6/1	6	--	1	5
3	Breast Cancers	48	17	4	14/2	5	1	5	6
4	Colorectal Cancer	18	5	--	7	6	--	--	3
5	Prostate Cancer	27	7	3	8/1	2	4	2	4
6	Liver Cancer	9	2	--	2/1	3	--	1	--
7	Bladder Cancer	6	1	--	4	--	--	1	--
8	Pancreatic Cancer	21	9	1	4/3	1	1	2	--
9	Ovarian Cancer	8	2	1	3/1	--	1	--	1
10	Endometrial Cancer	15	6	5	2	--	1	1	--
11	Head and Neck Cancers	10	2	2	3/1	2	--	--	2
12	Oral Cancers	8	--	2	4	1	--	1	2

Search keywords: Condition or disease: (Cancer/lung cancer/breast cancer/colorectal cancer/ prostate cancer/liver cancer/bladder cancer/pancreatic cancer/ovarian cancer/endometrial cancer/head and neck cancers/ oral cancers) + Other terms: (Metformin). Note: (1) The trials that use metformin may be studying one or more cancers, and hence, there will be an overlap in the numbers provided. (2) * Has results: Indicative of the trials that have results provided in https://clinicaltrials.gov/. For these studies and other studies where ‘no results’ have been posted on https://clinicaltrials.gov/, there may be publications/data that have not been reported in this website. (3) #Unknown Status: The study has crossed its proposed date of completion, but the status remains unverified for over 2 years. (4) These data were compiled on 29 June 2020.

**Table 3:** Status of metformin and drug/radiation-related clinical trials in breast cancers (https://clinicaltrials.gov/).

Serial No:	Metformin + Drug */Radiation	Total Number of Registered Trials	Completed	Active, Not Recruiting	Active, Recruiting	Terminated	Withdrawn/Suspended	Unknown Status #
1	5-fluorouracil (5-FU)	1	--	--	1	--	--	--
2	Carboplatin	1	--	0	1	--	--	--
3	Cyclophosphamide	5	1	1	3	--	--	--
4	Docetaxel	3	--	1	2	--	--	--
5	Doxorubicin/adriamycin	6	1	2	2	1	--	--
6	Epirubicin	1	--	--	1	--	--	--
7	Everolimus/Temsirolimus	2	1	--	--	1	--	--
8	Letrozole	2	1	--	1	--	--	--
9	Paclitaxel	5	--	2	1	1	--	1
10	Radiation/Radiotherapy	4	1	--	1	1	--	1

Search keywords: Condition or disease: Breast cancer + Other terms: Metformin, (5-fluorouracil; 5FU/Carboplatin/Cyclophosphamide/Docetaxel/Doxorubicin/Epirubicin/Everolimus/Temsirolimus/Letrozole/Paclitaxel/Radiation/Radiotherapy). Unknown Status: The study has crossed its proposed date of completion, but status remains unverified for over 2 years. * The drugs listed are the commonly administered chemotherapeutic agents in breast cancer. Note: (1) In the clinical trials, metformin is being used in combination with another drug/a combination of drugs, and hence, there will be an overlap in the numbers provided. (2) # Unknown Status: The study has crossed its proposed date of completion, but status remains unverified for over 2 years. (3) These data were compiled on 29 June 2020.

**Table 4:** Cancer stem cell (CSC)-related clinical trials involving metformin intervention (https://clinicaltrials.gov/).

Serial No:	Official Title	Trial Phase	Intervention Using Metformin	Objectives	Type of Cancer	Clinicaltrials.Gov ID (NCT Number)/Status, Actual Month and Year Related to the Status	Publications/References
1	Impact of Pretreatment with Metformin on Colorectal Cancer Stem Cells (CCSC) and Related Pharmacodynamic Parameters	Phase I	Metformin	Primary outcome measures: Expression of CD133 in tumors from patients treated with metformin in comparison to patients not treated with metformin	Colon Cancer	NCT01440127/Terminated October 2012	[299] (Abstract only)
2	A Phase II Evaluation of Metformin, Targeting Cancer Stem Cells for Prevention of Relapse in Patients with Stage IIC/III/IV Ovarian, Fallopian Tube, and Primary Peritoneal Cancer	Phase II	Metformin	Primary outcome measures: Recurrence-Free Survival Secondary outcome measures: Overall Survival	Ovarian, Fallopian Tube, and Primary Peritoneal Cancer	NCT01579812/Completed July 2017	[300]
3	A Pharmacodynamic Study of Metformin in Patients with Resectable Pancreatic Cancer	Phase I	Metformin hydrochloride	Primary outcome measures: Pancreatic tumor cell proliferation and apoptosis as measured by the percentage of Ki67 positive, percentage of TUNEL positive and mitotic counts in tissue samples. Secondary outcome measures: (1) Occurrence of grade 3 and 4 toxicities. (2) Expression of phospho-ACC and phospho-mTOR in tissue samples. (3) Percentage of pancreatic cancer stem cells in tissue samples.	Stage IA, IB, IIA, and IIB Pancreatic Cancer	NCT01954732/Completed March 2015	No results posted

Search keywords: Condition or disease: cancer + Other terms: metformin, cancer stem cells. Note: (1) The search yielded a list of 5 studies. Only three of the studies (mentioned in the table) have outcome measures that study the effect of metformin on CSCs, while the other two studies mention stem cells in their brief summary/work plan but do not have outcome measures that directly study the effect of metformin on CSCs. (2) These data were compiled on 29 June 2020.

## Abbreviations

5-FU	5-fluorouracil
ABC	ATP binding cassette
ABCB1	ABC sub-family B1; MDR1; P-gp
ABCB5	ABC sub-family B5
ABCC1	ABC sub-family C1
ABCC3	ABC sub-family C3
ABCG2	ABC sub-family G2; BCRP
ACC	Acetyl–CoA carboxylase
Akt	Protein kinase B
ALDH	Aldehyde dehydrogenase
AMPK	5′ adenosine monophosphate-activated protein kinase
BCRP	Breast cancer resistance protein; ABCG2
BCSCs	Breast cancer stem cells
BL1	Basal-like 1; TNBC subtype
BL2	Basal-like 2; TNBC subtype
CAFs	Cancer-associated fibroblasts
CCL2	Chemotactic protein 1
CSCs	Cancer stem cells
CTCs	Circulating tumor cells
CXCL7	Chemokine (C-X-C motif) ligand 7
CXCR1	Chemokine (C-X-C motif) receptor 1
DDIT4	DNA damage-inducible transcript 4
DICER	Double-stranded RNA specific endoribonuclease
DNMT	DNA-methyl transferase
DOX	Doxorubicin
DTCs	Disseminated tumor cells
DTX	Docetaxel
DVL3	Dishevelled 3
EGFR	Epidermal growth factor receptor
EMT	Epithelial-mesenchymal transition
Epi	Epirubicin
ER	Estrogen receptor
ERK	Extracellular signal-regulated kinase
FAC	5-FU, DOX and cyclophosphamide
FACS	Fluorescence activated cell sorter
FAK	Focal adhesion kinase
FEC	5-FU, epirubicin and cyclophosphamide
Gli	Glioma-associated oncogene homolog
GRP78	Glucose regulated protein 78
GSK-3β	Glycogen synthase kinase 3 beta
GST	Glutathione S-transferase
HER2	Human epidermal growth factor receptor 2
Hh	Hedgehog
HIF-1α/β	Hypoxia-inducible factor 1 alpha/beta
HMGB1	High-mobility group box 1
IC_50_	Half maximal inhibitory concentration
IGF-1	Insulin-like growth factor 1
IGF-1R	IGF-1 receptor
IL6/8	Interleukin 6/8
IM	Immunomodulatory; TNBC subtype
JAK	Janus kinase
KLF5	Krüppel-like factor 5
LAR	Luminal androgen receptor; TNBC subtype
M	Mesenchymal; TNBC subtype
MAPK	Mitogen activated protein kinase
MATE1/2	Multidrug and toxin extrusion protein 1 and 2
MCL1	Myeloid cell leukemia 1
MDR	Multi-drug resistance
MDR1	Multi-drug resistance protein 1; ABCB1; P-gp
MET	Mesenchymal-epithelial transition
miRNA	Micro-RNA
Mito	Mitomycin
MRP1	Multidrug resistance-associated protein 1; ABCC1
MSFE	Mammosphere forming efficiency
MSL	Mesenchymal stem-like; TNBC subtype
mTOR	Mammalian target of rapamycin
NF-κB	Nuclear factor-kappa B
NOD	Non-obese diabetic
NRF2	Nuclear erythroid-related factor 2
*O*-CMC	*O*-carboxymethyl chitosan
OCT1/2/3	Organic cation transporters 1, 2 and 3
OS	Overall survival
P-gp	P-glycoprotein; ABCB1; MDR1
PARP1	Poly (ADP-ribose) polymerase 1
PDGF-B	Platelet-derived growth factor B
PERK	Protein kinase RNA-like endoplasmic reticulum kinase
PFS	Progression free survival
PHGDH	Phosphoglycerate dehydrogenase
PI3K	Phosphatidylinositol-3-kinase
PKA	Protein kinase A
PLGA-PEG	Poly (lactic acid-co-glycolic acid)-polyethylene glycol
PMAT	Plasma membrane monoamine transporter
PR	Progesterone receptor
Ptch	Patched; Hh ligand receptor
PTX	Paclitaxel
RAD51	RecA homolog DNA repair recombinase
RANK-L	Receptor activator of nuclear factor-kappa B ligand
REDD1	Regulated DNA damage 1
RFS	Recurrence-free survival
SCID	Severe combined immunodeficiency
SHh	Sonic Hh
SMAD	Sma-and mothers against decapentaplegic homolog
Smo	Smoothened; Hh ligand co-receptor
SOX2	Sex determining region Y box transcription factor 2
STAT3	Signal transducer and activator of transcription 3
TAMs	Tumor associated macrophages
TFF1	Trefoil factor 1/protein pS2
TGFβ	Transforming growth factor beta
TNBCs	Triple negative breast cancers
TNFα	Tumor necrosis factor alpha
XIAP	X-linked inhibitor of apoptosis

## References

[1] WHO (2018). Cancer-Fact Sheets. https://www.who.int/news-room/fact-sheets/detail/cancer.

[2] Hawkes N. (2015). A comprehensive history of cancer treatment. https://www.raconteur.net/healthcare/history-of-cancer-treatment.

[3] ACS (2014). The History of Cancer-Cancer in the Sixteenth to Eighteenth Centuries. https://www.cancer.org/cancer/cancer-basics/history-of-cancer/sixteenth-to-eighteenth-centuries.html.

[4] Morrison W.B. (2010). Cancer Chemotherapy: An Annotated History. J. Vet. Intern. Med..

[5] DeVita V.T., Chu E. (2008). A History of Cancer Chemotherapy. Cancer Res..

[6] Schmidt F., Efferth T. (2016). Tumor Heterogeneity, Single-Cell Sequencing, and Drug Resistance. Pharmaceuticals.

[7] Chatterjee N., Bivona T.G. (2019). Polytherapy and Targeted Cancer Drug Resistance. Trends Cancer.

[8] McGranahan N., Swanton C. (2017). Clonal Heterogeneity and Tumor Evolution: Past, Present, and the Future. Cell.

[9] Konieczkowski D.J., Johannessen C.M., Garraway L.A. (2018). A Convergence-Based Framework for Cancer Drug Resistance. Cancer Cell.

[10] Gupta P.B., Pastushenko I., Skibinski A., Blanpain C., Kuperwasser C. (2019). Phenotypic Plasticity: Driver of Cancer Initiation, Progression, and Therapy Resistance. Cell Stem Cell.

[11] Muley H., Fadó R., Rodríguez-Rodríguez R., Casals N. (2020). Drug uptake-based chemoresistance in breast cancer treatment. Biochem. Pharmacol..

[12] Varghese E., Samuel S.M., Abotaleb M., Cheema S., Mamtani R., Büsselberg D. (2018). The “Yin and Yang” of Natural Compounds in Anticancer Therapy of Triple-Negative Breast Cancers. Cancers.

[13] Varghese E., Samuel S.M., Líšková A., Samec M., Kubatka P., Büsselberg D. (2020). Targeting Glucose Metabolism to Overcome Resistance to Anticancer Chemotherapy in Breast Cancer. Cancers.

[14] Subbiah S., Gopu G., Senthilkumar P., Muniasamy P. (2017). Molecular subtypes as a predictor of response to neoadjuvant chemotherapy in breast cancer patients. Indian J. Cancer.

[15] Lehmann B.D., Bauer J.A., Chen X., Sanders M.E., Chakravarthy A.B., Shyr Y., Pietenpol J.A. (2011). Identification of human triple-negative breast cancer subtypes and preclinical models for selection of targeted therapies. J. Clin. Investig..

[16] ACS (2019). Treatment of Breast Cancer by Stage. https://www.cancer.org/cancer/breast-cancer/treatment/treatment-of-breast-cancer-by-stage.html.

[17] Moiseenko F., Volkov N., Bogdanov A., Dubina M., Moiseyenko V. (2017). Resistance mechanisms to drug therapy in breast cancer and other solid tumors: An opinion. F1000Research.

[18] Tripathi L., Datta S.S., Agrawal S.K., Chatterjee S., Ahmed R. (2017). Stigma Perceived by Women Following Surgery for Breast Cancer. Indian J. Med. Paediatr Oncol..

[19] Vrinten C., Gallagher A., Waller J., Marlow L.A.V. (2019). Cancer stigma and cancer screening attendance: A population based survey in England. BMC Cancer.

[20] Rivera-Franco M.M., Leon-Rodriguez E. (2018). Delays in Breast Cancer Detection and Treatment in Developing Countries. Breast Cancer.

[21] Gottesman M.M. (2002). Mechanisms of Cancer Drug Resistance. Ann. Rev. Med..

[22] Ozakinci G., Sobota A., Humphris G. (2014). Fear of Cancer Recurrence Among Breast Cancer Survivors. Curr. Breast Cancer Rep..

[23] Ziner K.W., Sledge G.W., Bell C.J., Johns S., Miller K.D., Champion V.L. (2012). Predicting fear of breast cancer recurrence and self-efficacy in survivors by age at diagnosis. Oncol. Nurs. Forum.

[24] Van Maaren M.C., de Munck L., Strobbe L.J.A., Sonke G.S., Westenend P.J., Smidt M.L., Poortmans P.M.P., Siesling S. (2019). Ten-year recurrence rates for breast cancer subtypes in the Netherlands: A large population-based study. Int. J. Cancer.

[25] Shim H.J., Kim S.H., Kang B.J., Choi B.G., Kim H.S., Cha E.S., Song B.J. (2014). Breast cancer recurrence according to molecular subtype. Asian Pac. J. Cancer Prev..

[26] Montagna E., Bagnardi V., Rotmensz N., Viale G., Renne G., Cancello G., Balduzzi A., Scarano E., Veronesi P., Luini A. (2012). Breast cancer subtypes and outcome after local and regional relapse. Ann. Oncol..

[27] Lim E., Metzger-Filho O., Winer E.P. (2012). The natural history of hormone receptor-positive breast cancer. Oncology.

[28] Zimmerman M.P., Mehr S.R. (2014). Recurrence of Breast Cancer Years After the Initial Tumor. Am. J. Manag. Care.

[29] Coussens L.M., Werb Z. (2002). Inflammation and cancer. Nature.

[30] Seyfried T.N., Flores R.E., Poff A.M., D’Agostino D.P. (2014). Cancer as a metabolic disease: Implications for novel therapeutics. Carcinogenesis.

[31] De Martel C., Ferlay J., Franceschi S., Vignat J., Bray F., Forman D., Plummer M. (2012). Global burden of cancers attributable to infections in 2008: A review and synthetic analysis. Lancet Oncol..

[32] Van Elsland D., Neefjes J. (2018). Bacterial infections and cancer. EMBO Rep..

[33] Morales-Sánchez A., Fuentes-Pananá E.M. (2014). Human viruses and cancer. Viruses.

[34] Wang X., Zhang H., Chen X. (2019). Drug resistance and combating drug resistance in cancer. Cancer Drug Resist..

[35] Lovly C.M., Salama A.K.S., Salgia R. (2016). Tumor Heterogeneity and Therapeutic Resistance. Am. Soc. Clin. Oncol. Educ. Book.

[36] Dzobo K., Senthebane D.A., Thomford N.E., Rowe A., Dandara C., Parker M.I. (2018). Not Everyone Fits the Mold: Intratumor and Intertumor Heterogeneity and Innovative Cancer Drug Design and Development. Omics: A J. Integr. Biol..

[37] Chen W., Qin Y., Liu S. (2018). Cytokines, breast cancer stem cells (BCSCs) and chemoresistance. Clin. Transl. Med..

[38] Chuthapisith S., Eremin J., El-Sheemey M., Eremin O. (2010). Breast cancer chemoresistance: Emerging importance of cancer stem cells. Surg. Oncol..

[39] De Angelis M.L., Francescangeli F., Zeuner A. (2019). Breast Cancer Stem Cells as Drivers of Tumor Chemoresistance, Dormancy and Relapse: New Challenges and Therapeutic Opportunities. Cancers.

[40] Ji X., Lu Y., Tian H., Meng X., Wei M., Cho W.C. (2019). Chemoresistance mechanisms of breast cancer and their countermeasures. Biomed. Pharmacother..

[41] Ma L., Zong X. (2020). Metabolic Symbiosis in Chemoresistance: Refocusing the Role of Aerobic Glycolysis. Front. Oncol..

[42] Nunes T., Hamdan D., Leboeuf C., El Bouchtaoui M., Gapihan G., Nguyen T.T., Meles S., Angeli E., Ratajczak P., Lu H. (2018). Targeting Cancer Stem Cells to Overcome Chemoresistance. Int. J. Mol. Sci..

[43] Velaei K., Samadi N., Barazvan B., Soleimani Rad J. (2016). Tumor microenvironment-mediated chemoresistance in breast cancer. Breast.

[44] Housman G., Byler S., Heerboth S., Lapinska K., Longacre M., Snyder N., Sarkar S. (2014). Drug resistance in cancer: An overview. Cancers.

[45] Hu W., Tan C., He Y., Zhang G., Xu Y., Tang J. (2018). Functional miRNAs in breast cancer drug resistance. Onco Targets Ther..

[46] Nedeljković M., Damjanović A. (2019). Mechanisms of Chemotherapy Resistance in Triple-Negative Breast Cancer-How We Can Rise to the Challenge. Cells.

[47] Blendea M.C., Thompson M.J., Malkani S. (2010). Diabetes and Chronic Liver Disease: Etiology and Pitfalls in Monitoring. Clin. Diabetes.

[48] Leon B.M., Maddox T.M. (2015). Diabetes and cardiovascular disease: Epidemiology, biological mechanisms, treatment recommendations and future research. World J. Diabetes.

[49] Min T.Z., Stephens M.W., Kumar P., Chudleigh R.A. (2012). Renal complications of diabetes. Br. Med. Bull..

[50] Said G. (2007). Diabetic neuropathy--a review. Nat. Clin. Pract. Neurol..

[51] Samuel S.M., Varghese E., Kubatka P., Triggle C.R., Büsselberg D. (2019). Metformin: The Answer to Cancer in a Flower? Current Knowledge and Future Prospects of Metformin as an Anti-Cancer Agent in Breast Cancer. Biomolecules.

[52] Samuel S.M., Varghese E., Varghese S., Busselberg D. (2018). Challenges and perspectives in the treatment of diabetes associated breast cancer. Cancer Treat. Rev..

[53] Giovannucci E., Harlan D.M., Archer M.C., Bergenstal R.M., Gapstur S.M., Habel L.A., Pollak M., Regensteiner J.G., Yee D. (2010). Diabetes and cancer: A consensus report. Diabetes Care.

[54] Onitilo A.A., Engel J.M., Glurich I., Stankowski R.V., Williams G.M., Doi S.A. (2012). Diabetes and cancer I: Risk, survival, and implications for screening. Cancer Causes Control.

[55] Chowdhury T.A. (2010). Diabetes and cancer. QJM Mon. J. Assoc. Physicians.

[56] Ryu T.Y., Park J., Scherer P.E. (2014). Hyperglycemia as a risk factor for cancer progression. Diabetes Metab. J..

[57] Xu C.X., Zhu H.H., Zhu Y.M. (2014). Diabetes and cancer: Associations, mechanisms, and implications for medical practice. World J. Diabetes.

[58] Evans J.M., Donnelly L.A., Emslie-Smith A.M., Alessi D.R., Morris A.D. (2005). Metformin and reduced risk of cancer in diabetic patients. BMJ.

[59] De A., Kuppusamy G. (2019). Metformin in breast cancer: Preclinical and clinical evidence. Curr. Probl. Cancer.

[60] Chen E.C., Liang X., Yee S.W., Geier E.G., Stocker S.L., Chen L., Giacomini K.M. (2015). Targeted disruption of organic cation transporter 3 attenuates the pharmacologic response to metformin. Mol. Pharmacol..

[61] Chen Y., Teranishi K., Li S., Yee S.W., Hesselson S., Stryke D., Johns S.J., Ferrin T.E., Kwok P., Giacomini K.M. (2009). Genetic variants in multidrug and toxic compound extrusion-1, hMATE1, alter transport function. Pharm. J..

[62] Choi M.K., Jin Q.R., Jin H.E., Shim C.K., Cho D.Y., Shin J.G., Song I.S. (2007). Effects of tetraalkylammonium compounds with different affinities for organic cation transporters on the pharmacokinetics of metformin. Biopharm. Drug Dispos..

[63] Kimura N., Masuda S., Tanihara Y., Ueo H., Okuda M., Katsura T., Inui K. (2005). Metformin is a superior substrate for renal organic cation transporter OCT2 rather than hepatic OCT1. Drug Metab. Pharmacokinet..

[64] Li S., Chen Y., Zhang S., More S.S., Huang X., Giacomini K.M. (2011). Role of organic cation transporter 1, OCT1 in the pharmacokinetics and toxicity of cis-diammine(pyridine)chloroplatinum(II) and oxaliplatin in mice. Pharm. Res..

[65] Liang X., Giacomini K.M. (2017). Transporters Involved in Metformin Pharmacokinetics and Treatment Response. J. Pharm. Sci..

[66] Masuda S., Terada T., Yonezawa A., Tanihara Y., Kishimoto K., Katsura T., Ogawa O., Inui K. (2006). Identification and functional characterization of a new human kidney-specific H+/organic cation antiporter, kidney-specific multidrug and toxin extrusion 2. J. Am. Soc. Nephrol. JASN.

[67] Zhou M., Xia L., Wang J. (2007). Metformin transport by a newly cloned proton-stimulated organic cation transporter (plasma membrane monoamine transporter) expressed in human intestine. Drug Metab. Dispos..

[68] Liang X., Chien H.C., Yee S.W., Giacomini M.M., Chen E.C., Piao M., Hao J., Twelves J., Lepist E.I., Ray A.S. (2015). Metformin Is a Substrate and Inhibitor of the Human Thiamine Transporter, THTR-2 (SLC19A3). Mol. Pharm..

[69] Shu Y., Brown C., Castro R.A., Shi R.J., Lin E.T., Owen R.P., Sheardown S.A., Yue L., Burchard E.G., Brett C.M. (2008). Effect of genetic variation in the organic cation transporter 1, OCT1, on metformin pharmacokinetics. Clin. Pharm. Ther..

[70] Shu Y., Sheardown S.A., Brown C., Owen R.P., Zhang S., Castro R.A., Ianculescu A.G., Yue L., Lo J.C., Burchard E.G. (2007). Effect of genetic variation in the organic cation transporter 1 (OCT1) on metformin action. J. Clin. Investig..

[71] Cai H., Zhang Y., Han T.K., Everett R.S., Thakker D.R. (2016). Cation-selective transporters are critical to the AMPK-mediated antiproliferative effects of metformin in human breast cancer cells. Int. J. Cancer.

[72] Checkley L.A., Rudolph M.C., Wellberg E.A., Giles E.D., Wahdan-Alaswad R.S., Houck J.A., Edgerton S.M., Thor A.D., Schedin P., Anderson S.M. (2017). Metformin Accumulation Correlates with Organic Cation Transporter 2 Protein Expression and Predicts Mammary Tumor Regression in Vivo. Cancer Prev. Res..

[73] Cai H., Everett R.S., Thakker D.R. (2019). Efficacious dose of metformin for breast cancer therapy is determined by cation transporter expression in tumours. Br. J. Pharmacol..

[74] Chowdhury S., Yung E., Pintilie M., Muaddi H., Chaib S., Yeung M., Fusciello M., Sykes J., Pitcher B., Hagenkort A. (2016). MATE2 Expression Is Associated with Cancer Cell Response to Metformin. PLoS ONE.

[75] Correia S., Carvalho C., Santos M.S., Seica R., Oliveira C.R., Moreira P.I. (2008). Mechanisms of action of metformin in type 2 diabetes and associated complications: An overview. Mini Rev. Med. Chem..

[76] Rahmani J., Manzari N., Thompson J., Gudi S.K., Chhabra M., Naik G., Mousavi S.M., Varkaneh H.K., Clark C., Zhang Y. (2019). The effect of metformin on biomarkers associated with breast cancer outcomes: A systematic review, meta-analysis, and dose-response of randomized clinical trials. Clin. Transl. Oncol..

[77] Ferroni P., Riondino S., Buonomo O., Palmirotta R., Guadagni F., Roselli M. (2015). Type 2 Diabetes and Breast Cancer: The Interplay between Impaired Glucose Metabolism and Oxidant Stress. Oxid. Med. Cell Longev..

[78] Rojas L.B., Gomes M.B. (2013). Metformin: An old but still the best treatment for type 2 diabetes. Diabetol. Metab. Syndr..

[79] Triggle C.R., Ding H. (2017). Metformin is not just an antihyperglycaemic drug but also has protective effects on the vascular endothelium. Acta Physiol..

[80] Sośnicki S., Kapral M., Węglarz L. (2016). Molecular targets of metformin antitumor action. Pharmacol. Rep..

[81] Warburg O., Wind F., Negelein E. (1927). THE METABOLISM OF TUMORS IN THE BODY. J. Gen. Physiol..

[82] Le A., Lane A.N., Hamaker M., Bose S., Gouw A., Barbi J., Tsukamoto T., Rojas C.J., Slusher B.S., Zhang H. (2012). Glucose-Independent Glutamine Metabolism via TCA Cycling for Proliferation and Survival in B Cells. Cell Metab..

[83] Metallo C.M., Gameiro P.A., Bell E.L., Mattaini K.R., Yang J., Hiller K., Jewell C.M., Johnson Z.R., Irvine D.J., Guarente L. (2012). Reductive glutamine metabolism by IDH1 mediates lipogenesis under hypoxia. Nature.

[84] Samuel S.M., Ghosh S., Majeed Y., Arunachalam G., Emara M.M., Ding H., Triggle C.R. (2017). Metformin represses glucose starvation induced autophagic response in microvascular endothelial cells and promotes cell death. Biochem. Pharmacol..

[85] Daugan M., Dufaÿ Wojcicki A., d’Hayer B., Boudy V. (2016). Metformin: An anti-diabetic drug to fight cancer. Pharmacol. Res..

[86] Hawley S.A., Gadalla A.E., Olsen G.S., Hardie D.G. (2002). The Antidiabetic Drug Metformin Activates the AMP-Activated Protein Kinase Cascade via an Adenine Nucleotide-Independent Mechanism. Diabetes.

[87] Zhou G., Myers R., Li Y., Chen Y., Shen X., Fenyk-Melody J., Wu M., Ventre J., Doebber T., Fujii N. (2001). Role of AMP-activated protein kinase in mechanism of metformin action. J. Clin. Investig..

[88] Meng S., Cao J., He Q., Xiong L., Chang E., Radovick S., Wondisford F.E., He L. (2015). Metformin activates AMP-activated protein kinase by promoting formation of the αβγ heterotrimeric complex. J. Biol. Chem..

[89] Howell J.J., Hellberg K., Turner M., Talbott G., Kolar M.J., Ross D.S., Hoxhaj G., Saghatelian A., Shaw R.J., Manning B.D. (2017). Metformin Inhibits Hepatic mTORC1 Signaling via Dose-Dependent Mechanisms Involving AMPK and the TSC Complex. Cell Metab..

[90] Sabnis H.S., Somasagara R.R., Bunting K.D. (2017). Targeting MYC Dependence by Metabolic Inhibitors in Cancer. Genes.

[91] Shen P., Reineke L.C., Knutsen E., Chen M., Pichler M., Ling H., Calin G.A. (2018). Metformin blocks MYC protein synthesis in colorectal cancer via mTOR-4EBP-eIF4E and MNK1-eIF4G-eIF4E signaling. Mol. Oncol..

[92] Hattori Y., Suzuki K., Hattori S., Kasai K. (2006). Metformin Inhibits Cytokine-Induced Nuclear Factor κB Activation Via AMP-Activated Protein Kinase Activation in Vascular Endothelial Cells. Hypertension.

[93] Sekino N., Kano M., Matsumoto Y., Sakata H., Akutsu Y., Hanari N., Murakami K., Toyozumi T., Takahashi M., Otsuka R. (2018). Antitumor effects of metformin are a result of inhibiting nuclear factor kappa B nuclear translocation in esophageal squamous cell carcinoma. Cancer Sci..

[94] Xu S., Yang Z., Jin P., Yang X., Li X., Wei X., Wang Y., Long S., Zhang T., Chen G. (2018). Metformin Suppresses Tumor Progression by Inactivating Stromal Fibroblasts in Ovarian Cancer. Mol. Cancer Ther..

[95] Li P., Zhao M., Parris A., Feng X., Yang X. (2015). P53 is required for metformin-induced growth inhibition, senescence and apoptosis in breast cancer cells. Biochem. Biophys. Res. Commun..

[96] Chen L., Ahmad N., Liu X. (2016). Combining p53 stabilizers with metformin induces synergistic apoptosis through regulation of energy metabolism in castration-resistant prostate cancer. Cell Cycle.

[97] Yi Y., Zhang W., Yi J., Xiao Z.-X. (2019). Role of p53 Family Proteins in Metformin Anti-Cancer Activities. J. Cancer.

[98] Yi G., He Z., Zhou X., Xian L., Yuan T., Jia X., Hong J., He L., Liu J. (2013). Low concentration of metformin induces a p53-dependent senescence in hepatoma cells via activation of the AMPK pathway. Int. J. Oncol..

[99] Noren Hooten N., Martin-Montalvo A., Dluzen D.F., Zhang Y., Bernier M., Zonderman A.B., Becker K.G., Gorospe M., de Cabo R., Evans M.K. (2016). Metformin-mediated increase in DICER1 regulates microRNA expression and cellular senescence. Aging Cell.

[100] Pulito C., Donzelli S., Muti P., Puzzo L., Strano S., Blandino G. (2014). microRNAs and cancer metabolism reprogramming: The paradigm of metformin. Ann. Transl. Med..

[101] Blandino G., Valerio M., Cioce M., Mori F., Casadei L., Pulito C., Sacconi A., Biagioni F., Cortese G., Galanti S. (2012). Metformin elicits anticancer effects through the sequential modulation of DICER and c-MYC. Nat. Commun..

[102] Lumachi F., Brunello A., Maruzzo M., Basso U., Basso S.M. (2013). Treatment of estrogen receptor-positive breast cancer. Curr. Med. Chem..

[103] Ma J., Guo Y., Chen S., Zhong C., Xue Y., Zhang Y., Lai X., Wei Y., Yu S., Zhang J. (2014). Metformin enhances tamoxifen-mediated tumor growth inhibition in ER-positive breast carcinoma. BMC Cancer.

[104] Kim J., Lee J., Jang S.Y., Kim C., Choi Y., Kim A. (2016). Anticancer effect of metformin on estrogen receptor-positive and tamoxifen-resistant breast cancer cell lines. Oncol. Rep..

[105] Giles E.D., Jindal S., Wellberg E.A., Schedin T., Anderson S.M., Thor A.D., Edwards D.P., MacLean P.S., Schedin P. (2018). Metformin inhibits stromal aromatase expression and tumor progression in a rodent model of postmenopausal breast cancer. Breast Cancer Res. BCR.

[106] Zhao Y., Gong C., Wang Z., Zhang J., Wang L., Zhang S., Cao J., Tao Z., Li T., Wang B. (2017). A randomized phase II study of aromatase inhibitors plus metformin in pre-treated postmenopausal patients with hormone receptor positive metastatic breast cancer. Oncotarget.

[107] Wang J.-C., Li G.-Y., Wang B., Han S.-X., Sun X., Jiang Y.-N., Shen Y.-W., Zhou C., Feng J., Lu S.-Y. (2019). Metformin inhibits metastatic breast cancer progression and improves chemosensitivity by inducing vessel normalization via PDGF-B downregulation. J. Exp. Clin. Cancer Res..

[108] Shafiei-Irannejad V., Samadi N., Yousefi B., Salehi R., Velaei K., Zarghami N. (2018). Metformin enhances doxorubicin sensitivity via inhibition of doxorubicin efflux in P-gp-overexpressing MCF-7 cells. Chem. Biol. Drug Des..

[109] Shafiei-Irannejad V., Samadi N., Salehi R., Yousefi B., Rahimi M., Akbarzadeh A., Zarghami N. (2018). Reversion of Multidrug Resistance by Co-Encapsulation of Doxorubicin and Metformin in Poly(lactide-co-glycolide)-d-α-tocopheryl Polyethylene Glycol 1000 Succinate Nanoparticles. Pharm. Res..

[110] Rao M., Gao C., Guo M., Law B.Y.K., Xu Y. (2018). Effects of metformin treatment on radiotherapy efficacy in patients with cancer and diabetes: A systematic review and meta-analysis. Cancer Manag. Res..

[111] Davies G., Lobanova L., Dawicki W., Groot G., Gordon J.R., Bowen M., Harkness T., Arnason T. (2017). Metformin inhibits the development, and promotes the resensitization, of treatment-resistant breast cancer. PLoS ONE.

[112] Ariaans G., Jalving M., Vries E.G.E.d., Jong S.d. (2017). Anti-tumor effects of everolimus and metformin are complementary and glucose-dependent in breast cancer cells. BMC Cancer.

[113] Deng J., Peng M., Wang Z., Zhou S., Xiao D., Deng J., Yang X., Peng J., Yang X. (2019). Novel application of metformin combined with targeted drugs on anticancer treatment. Cancer Sci..

[114] Liu B., Fan Z., Edgerton S.M., Yang X., Lind S.E., Thor A.D. (2011). Potent anti-proliferative effects of metformin on trastuzumab-resistant breast cancer cells via inhibition of erbB2/IGF-1 receptor interactions. Cell Cycle.

[115] Zeglinski M., Ludke A., Jassal D.S., Singal P.K. (2011). Trastuzumab-induced cardiac dysfunction: A ‘dual-hit’. Exp. Clin. Cardiol..

[116] Smith T.A., Phyu S.M., Akabuogu E.U. (2016). Effects of Administered Cardioprotective Drugs on Treatment Response of Breast Cancer Cells. Anticancer Res..

[117] Vázquez-Martín A., Oliveras-Ferraros C., del Barco S., Martín-Castillo B., Menéndez J.A. (2009). mTOR inhibitors and the anti-diabetic biguanide metformin: New insights into the molecular management of breast cancer resistance to the HER2 tyrosine kinase inhibitor lapatinib (Tykerb). Clin. Transl. Oncol..

[118] Chung Y.-C., Chang C.-M., Wei W.-C., Chang T.-W., Chang K.-J., Chao W.-T. (2018). Metformin-induced caveolin-1 expression promotes T-DM1 drug efficacy in breast cancer cells. Sci. Rep..

[119] Dasari S., Tchounwou P.B. (2014). Cisplatin in cancer therapy: Molecular mechanisms of action. Eur. J. Pharmacol..

[120] Lee J.O., Kang M.J., Byun W.S., Kim S.A., Seo I.H., Han J.A., Moon J.W., Kim J.H., Kim S.J., Lee E.J. (2019). Metformin overcomes resistance to cisplatin in triple-negative breast cancer (TNBC) cells by targeting RAD51. Breast Cancer Res..

[121] Tsai M.S., Kuo Y.H., Chiu Y.F., Su Y.C., Lin Y.W. (2010). Down-regulation of Rad51 expression overcomes drug resistance to gemcitabine in human non-small-cell lung cancer cells. J. Pharmacol. Exp. Ther..

[122] Quiros S., Roos W.P., Kaina B. (2011). Rad51 and BRCA2 - New Molecular Targets for Sensitizing Glioma Cells to Alkylating Anticancer Drugs. PLoS ONE.

[123] Qu C., Zhang W., Zheng G., Zhang Z., Yin J., He Z. (2014). Metformin reverses multidrug resistance and epithelial-mesenchymal transition (EMT) via activating AMP-activated protein kinase (AMPK) in human breast cancer cells. Mol. Cell Biochem..

[124] Zheng G., Peng F., Ding R., Yu Y., Ouyang Y., Chen Z., Xiao Z., He Z. (2010). Identification of proteins responsible for the multiple drug resistance in 5-fluorouracil-induced breast cancer cell using proteomics analysis. J. Cancer Res. Clin. Oncol..

[125] Zheng G., Xiong Y., Yi S., Zhang W., Peng B., Zhang Q., He Z. (2012). 14-3-3σ regulation by p53 mediates a chemotherapy response to 5-fluorouracil in MCF-7 breast cancer cells via Akt inactivation. FEBS Lett..

[126] Zhang W., Feng M., Zheng G., Chen Y., Wang X., Pen B., Yin J., Yu Y., He Z. (2012). Chemoresistance to 5-fluorouracil induces epithelial-mesenchymal transition via up-regulation of Snail in MCF7 human breast cancer cells. Biochem. Biophys. Res. Commun..

[127] Soo J.S., Ng C.H., Tan S.H., Malik R.A., Teh Y.C., Tan B.S., Ho G.F., See M.H., Taib N.A., Yip C.H. (2015). Metformin synergizes 5-fluorouracil, epirubicin, and cyclophosphamide (FEC) combination therapy through impairing intracellular ATP production and DNA repair in breast cancer stem cells. Apoptosis.

[128] Lobo N.A., Shimono Y., Qian D., Clarke M.F. (2007). The Biology of Cancer Stem Cells. Ann. Rev. Cell Dev. Biol..

[129] Pierce G.B., Wallace C. (1971). Differentiation of Malignant to Benign Cells. Cancer Res..

[130] Lapidot T., Sirard C., Vormoor J., Murdoch B., Hoang T., Caceres-Cortes J., Minden M., Paterson B., Caligiuri M.A., Dick J.E. (1994). A cell initiating human acute myeloid leukaemia after transplantation into SCID mice. Nature.

[131] Al-Hajj M., Wicha M.S., Benito-Hernandez A., Morrison S.J., Clarke M.F. (2003). Prospective identification of tumorigenic breast cancer cells. Proc. Natl. Acad. Sci. USA.

[132] Hurt E.M., Kawasaki B.T., Klarmann G.J., Thomas S.B., Farrar W.L. (2008). CD44+ CD24(-) prostate cells are early cancer progenitor/stem cells that provide a model for patients with poor prognosis. Br. J. Cancer.

[133] Patrawala L., Calhoun T., Schneider-Broussard R., Li H., Bhatia B., Tang S., Reilly J.G., Chandra D., Zhou J., Claypool K. (2006). Highly purified CD44+ prostate cancer cells from xenograft human tumors are enriched in tumorigenic and metastatic progenitor cells. Oncogene.

[134] Singh S.K., Hawkins C., Clarke I.D., Squire J.A., Bayani J., Hide T., Henkelman R.M., Cusimano M.D., Dirks P.B. (2004). Identification of human brain tumour initiating cells. Nature.

[135] Ricci-Vitiani L., Lombardi D.G., Pilozzi E., Biffoni M., Todaro M., Peschle C., De Maria R. (2007). Identification and expansion of human colon-cancer-initiating cells. Nature.

[136] Li C., Heidt D.G., Dalerba P., Burant C.F., Zhang L., Adsay V., Wicha M., Clarke M.F., Simeone D.M. (2007). Identification of Pancreatic Cancer Stem Cells. Cancer Res..

[137] Herschkowitz J.I. (2010). Breast cancer stem cells: Initiating a new sort of thinking. Dis. Models Mech..

[138] Velasco-Velázquez M.A., Homsi N., De La Fuente M., Pestell R.G. (2012). Breast cancer stem cells. Int. J. Biochem. Cell Biol..

[139] Ginestier C., Hur M.H., Charafe-Jauffret E., Monville F., Dutcher J., Brown M., Jacquemier J., Viens P., Kleer C.G., Liu S. (2007). ALDH1 is a marker of normal and malignant human mammary stem cells and a predictor of poor clinical outcome. Cell Stem Cell.

[140] Morel A.-P., Lièvre M., Thomas C., Hinkal G., Ansieau S., Puisieux A. (2008). Generation of breast cancer stem cells through epithelial-mesenchymal transition. PLoS ONE.

[141] Mani S.A., Guo W., Liao M.-J., Eaton E.N., Ayyanan A., Zhou A.Y., Brooks M., Reinhard F., Zhang C.C., Shipitsin M. (2008). The epithelial-mesenchymal transition generates cells with properties of stem cells. Cell.

[142] Ponti D., Costa A., Zaffaroni N., Pratesi G., Petrangolini G., Coradini D., Pilotti S., Pierotti M.A., Daidone M.G. (2005). Isolation and *In vitro* Propagation of Tumorigenic Breast Cancer Cells with Stem/Progenitor Cell Properties. Cancer Res..

[143] Visvader J.E., Lindeman G.J. (2008). Cancer stem cells in solid tumours: Accumulating evidence and unresolved questions. Nat. Rev. Cancer.

[144] Alison M.R., Lim S.M.L., Nicholson L.J. (2011). Cancer stem cells: Problems for therapy?. J. Pathol..

[145] Dean M., Fojo T., Bates S. (2005). Tumour stem cells and drug resistance. Nat. Rev. Cancer.

[146] Auffinger B., Tobias A.L., Han Y., Lee G., Guo D., Dey M., Lesniak M.S., Ahmed A.U. (2014). Conversion of differentiated cancer cells into cancer stem-like cells in a glioblastoma model after primary chemotherapy. Cell Death Differ..

[147] Hamerlik P., Lathia J.D., Rasmussen R., Wu Q., Bartkova J., Lee M., Moudry P., Bartek J., Fischer W., Lukas J. (2012). Autocrine VEGF-VEGFR2-Neuropilin-1 signaling promotes glioma stem-like cell viability and tumor growth. J. Exp. Med..

[148] Shien K., Toyooka S., Yamamoto H., Soh J., Jida M., Thu K.L., Hashida S., Maki Y., Ichihara E., Asano H. (2013). Acquired resistance to EGFR inhibitors is associated with a manifestation of stem cell-like properties in cancer cells. Cancer Res..

[149] Martins-Neves S.R., Cleton-Jansen A.-M., Gomes C.M.F. (2018). Therapy-induced enrichment of cancer stem-like cells in solid human tumors: Where do we stand?. Pharmacol. Res..

[150] Gao X., Sishc B.J., Nelson C.B., Hahnfeldt P., Bailey S.M., Hlatky L. (2016). Radiation-Induced Reprogramming of Pre-Senescent Mammary Epithelial Cells Enriches Putative CD44+/CD24−/low Stem Cell Phenotype. Front. Oncol..

[151] Debeb B.G., Xu W., Woodward W.A. (2009). Radiation Resistance of Breast Cancer Stem Cells: Understanding the Clinical Framework. J. Mammary Gland Biol. Neoplasia.

[152] Li F., Zhou K., Gao L., Zhang B., Li W., Yan W., Song X., Yu H., Wang S., Yu N. (2016). Radiation induces the generation of cancer stem cells: A novel mechanism for cancer radioresistance. Oncol. Lett..

[153] Zielske S.P., Spalding A.C., Wicha M.S., Lawrence T.S. (2011). Ablation of breast cancer stem cells with radiation. Transl. Oncol..

[154] Vidal S.J., Rodriguez-Bravo V., Galsky M., Cordon-Cardo C., Domingo-Domenech J. (2014). Targeting cancer stem cells to suppress acquired chemotherapy resistance. Oncogene.

[155] Chen X., Liao R., Li D., Sun J. (2017). Induced cancer stem cells generated by radiochemotherapy and their therapeutic implications. Oncotarget.

[156] Mukherjee P., Gupta A., Chattopadhyay D., Chatterji U. (2017). Modulation of SOX2 expression delineates an end-point for paclitaxel-effectiveness in breast cancer stem cells. Sci. Rep..

[157] Zhou J., Chen Q., Zou Y., Chen H., Qi L., Chen Y. (2019). Stem Cells and Cellular Origins of Breast Cancer: Updates in the Rationale, Controversies, and Therapeutic Implications. Front. Oncol..

[158] De Angelis M.L., Francescangeli F., La Torre F., Zeuner A. (2019). Stem Cell Plasticity and Dormancy in the Development of Cancer Therapy Resistance. Front. Oncol..

[159] Phi L.T.H., Sari I.N., Yang Y.-G., Lee S.-H., Jun N., Kim K.S., Lee Y.K., Kwon H.Y. (2018). Cancer Stem Cells (CSCs) in Drug Resistance and their Therapeutic Implications in Cancer Treatment. Stem Cells Int..

[160] Fluegen G., Avivar-Valderas A., Wang Y., Padgen M.R., Williams J.K., Nobre A.R., Calvo V., Cheung J.F., Bravo-Cordero J.J., Entenberg D. (2017). Phenotypic heterogeneity of disseminated tumour cells is preset by primary tumour hypoxic microenvironments. Nat. Cell Biol..

[161] Kim H., Lin Q., Glazer P.M., Yun Z. (2018). The hypoxic tumor microenvironment in vivo selects the cancer stem cell fate of breast cancer cells. Breast Cancer Res. BCR.

[162] Harrison H., Rogerson L., Gregson H.J., Brennan K.R., Clarke R.B., Landberg G. (2013). Contrasting Hypoxic Effects on Breast Cancer Stem Cell Hierarchy Is Dependent on ER-α Status. Cancer Res..

[163] Peppicelli S., Andreucci E., Ruzzolini J., Laurenzana A., Margheri F., Fibbi G., Del Rosso M., Bianchini F., Calorini L. (2017). The acidic microenvironment as a possible niche of dormant tumor cells. Cell. Mol. Life Sci..

[164] Linde N., Fluegen G., Aguirre-Ghiso J.A. (2016). The Relationship Between Dormant Cancer Cells and Their Microenvironment. Adv. Cancer Res..

[165] Ghiabi P., Jiang J., Pasquier J., Maleki M., Abu-Kaoud N., Rafii S., Rafii A. (2014). Endothelial cells provide a notch-dependent pro-tumoral niche for enhancing breast cancer survival, stemness and pro-metastatic properties. PLoS ONE.

[166] Comerford K.M., Wallace T.J., Karhausen J., Louis N.A., Montalto M.C., Colgan S.P. (2002). Hypoxia-inducible factor-1-dependent regulation of the multidrug resistance (MDR1) gene. Cancer Res..

[167] Sansone P., Ceccarelli C., Berishaj M., Chang Q., Rajasekhar V.K., Perna F., Bowman R.L., Vidone M., Daly L., Nnoli J. (2016). Self-renewal of CD133(hi) cells by IL6/Notch3 signalling regulates endocrine resistance in metastatic breast cancer. Nat. Commun..

[168] Luo M., Wicha M.S. (2015). Metabolic plasticity of cancer stem cells. Oncotarget.

[169] Fiorillo M., Sotgia F., Lisanti M.P. (2019). “Energetic” Cancer Stem Cells (e-CSCs): A New Hyper-Metabolic and Proliferative Tumor Cell Phenotype, Driven by Mitochondrial Energy. Front. Oncol..

[170] Jiang B. (2017). Aerobic glycolysis and high level of lactate in cancer metabolism and microenvironment. Genes Dis..

[171] Thiery J.P., Acloque H., Huang R.Y.J., Nieto M.A. (2009). Epithelial-Mesenchymal Transitions in Development and Disease. Cell.

[172] Hong D., Fritz A.J., Zaidi S.K., van Wijnen A.J., Nickerson J.A., Imbalzano A.N., Lian J.B., Stein J.L., Stein G.S. (2018). Epithelial-to-mesenchymal transition and cancer stem cells contribute to breast cancer heterogeneity. J. Cell Physiol..

[173] Kalluri R., Weinberg R.A. (2009). The basics of epithelial-mesenchymal transition. J. Clin. Investig..

[174] Zeisberg M., Neilson E.G. (2009). Biomarkers for epithelial-mesenchymal transitions. J. Clin. Investig..

[175] Loh C.-Y., Chai J.Y., Tang T.F., Wong W.F., Sethi G., Shanmugam M.K., Chong P.P., Looi C.Y. (2019). The E-Cadherin and N-Cadherin Switch in Epithelial-to-Mesenchymal Transition: Signaling, Therapeutic Implications, and Challenges. Cells.

[176] Krawczyk N., Meier-Stiegen F., Banys M., Neubauer H., Ruckhaeberle E., Fehm T. (2014). Expression of Stem Cell and Epithelial-Mesenchymal Transition Markers in Circulating Tumor Cells of Breast Cancer Patients. BioMed Res. Int..

[177] Christowitz C., Davis T., Isaacs A., van Niekerk G., Hattingh S., Engelbrecht A.-M. (2019). Mechanisms of doxorubicin-induced drug resistance and drug resistant tumour growth in a murine breast tumour model. BMC Cancer.

[178] Chen W.-C., Lai Y.-A., Lin Y.-C., Ma J.-W., Huang L.-F., Yang N.-S., Ho C.-T., Kuo S.-C., Way T.-D. (2013). Curcumin Suppresses Doxorubicin-Induced Epithelial–Mesenchymal Transition via the Inhibition of TGF-β and PI3K/AKT Signaling Pathways in Triple-Negative Breast Cancer Cells. J. Agric. Food Chem..

[179] Kim R.-K., Kaushik N., Suh Y., Yoo K.-C., Cui Y.-H., Kim M.-J., Lee H.-J., Kim I.-G., Lee S.-J. (2016). Radiation driven epithelial-mesenchymal transition is mediated by Notch signaling in breast cancer. Oncotarget.

[180] Hugo H., Ackland M.L., Blick T., Lawrence M.G., Clements J.A., Williams E.D., Thompson E.W. (2007). Epithelial—mesenchymal and mesenchymal—epithelial transitions in carcinoma progression. J. Cell Physiol..

[181] Yao D., Dai C., Peng S. (2011). Mechanism of the Mesenchymal–Epithelial Transition and Its Relationship with Metastatic Tumor Formation. Mol. Cancer Res..

[182] Luo M., Brooks M., Wicha M.S. (2015). Epithelial-mesenchymal plasticity of breast cancer stem cells: Implications for metastasis and therapeutic resistance. Curr. Pharm. Des..

[183] Thiery J.P. (2002). Epithelial–mesenchymal transitions in tumour progression. Nat. Rev. Cancer.

[184] Prieto-Vila M., Takahashi R.-U., Usuba W., Kohama I., Ochiya T. (2017). Drug Resistance Driven by Cancer Stem Cells and Their Niche. Int. J. Mol. Sci..

[185] Sridharan S., Howard C.M., Tilley A.M.C., Subramaniyan B., Tiwari A.K., Ruch R.J., Raman D. (2019). Novel and Alternative Targets Against Breast Cancer Stemness to Combat Chemoresistance. Front. Oncol..

[186] Leccia F., Del Vecchio L., Mariotti E., Di Noto R., Morel A.-P., Puisieux A., Salvatore F., Ansieau S. (2014). ABCG2, a novel antigen to sort luminal progenitors of BRCA1- breast cancer cells. Mol. Cancer.

[187] Tiezzi D.G., Sicchieri R.D., Mouro L.R., Oliveira T.M.G., Silveira W.A., Antonio H.M.R., Muglia V.F., de Andrade J.M. (2013). ABCG2 as a potential cancer stem cell marker in breast cancer. J. Clin. Oncol..

[188] Britton K.M., Eyre R., Harvey I.J., Stemke-Hale K., Browell D., Lennard T.W.J., Meeson A.P. (2012). Breast cancer, side population cells and ABCG2 expression. Cancer Lett..

[189] Arumugam A., Subramani R., Nandy S.B., Terreros D., Dwivedi A.K., Saltzstein E., Lakshmanaswamy R. (2019). Silencing growth hormone receptor inhibits estrogen receptor negative breast cancer through ATP-binding cassette sub-family G member 2. Exp. Mol. Med..

[190] Balaji S.A., Udupa N., Chamallamudi M.R., Gupta V., Rangarajan A. (2016). Role of the Drug Transporter ABCC3 in Breast Cancer Chemoresistance. PLoS ONE.

[191] Jiang Z.-S., Sun Y.-Z., Wang S.-M., Ruan J.-S. (2017). Epithelial-mesenchymal transition: Potential regulator of ABC transporters in tumor progression. J. Cancer.

[192] Dittmer J. (2018). Breast cancer stem cells: Features, key drivers and treatment options. Semin. Cancer Biol..

[193] Saxena M., Stephens M.A., Pathak H., Rangarajan A. (2011). Transcription factors that mediate epithelial-mesenchymal transition lead to multidrug resistance by upregulating ABC transporters. Cell Death Dis..

[194] Zhu Y., Yu F., Jiao Y., Feng J., Tang W., Yao H., Gong C., Chen J., Su F., Zhang Y. (2011). Reduced miR-128 in breast tumor-initiating cells induces chemotherapeutic resistance via Bmi-1 and ABCC5. Clin. Cancer Res..

[195] Gao M., Miao L., Liu M., Li C., Yu C., Yan H., Yin Y., Wang Y., Qi X., Ren J. (2016). miR-145 sensitizes breast cancer to doxorubicin by targeting multidrug resistance-associated protein-1. Oncotarget.

[196] Liang Z., Wu H., Xia J., Li Y., Zhang Y., Huang K., Wagar N., Yoon Y., Cho H.T., Scala S. (2010). Involvement of miR-326 in chemotherapy resistance of breast cancer through modulating expression of multidrug resistance-associated protein 1. Biochem. Pharmacol..

[197] Turdo A., Veschi V., Gaggianesi M., Chinnici A., Bianca P., Todaro M., Stassi G. (2019). Meeting the Challenge of Targeting Cancer Stem Cells. Front. Cell Dev. Biol..

[198] Shafee N., Smith C.R., Wei S., Kim Y., Mills G.B., Hortobagyi G.N., Stanbridge E.J., Lee E.Y.H.P. (2008). Cancer stem cells contribute to cisplatin resistance in Brca1/p53-mediated mouse mammary tumors. Cancer Res..

[199] Del Vecchio C.A., Feng Y., Sokol E.S., Tillman E.J., Sanduja S., Reinhardt F., Gupta P.B. (2014). De-differentiation confers multidrug resistance via noncanonical PERK-Nrf2 signaling. Plos. Biol..

[200] Phillips T.M., McBride W.H., Pajonk F. (2006). The response of CD24(-/low)/CD44+ breast cancer-initiating cells to radiation. J. Natl. Cancer Inst..

[201] Diehn M., Cho R.W., Lobo N.A., Kalisky T., Dorie M.J., Kulp A.N., Qian D., Lam J.S., Ailles L.E., Wong M. (2009). Association of reactive oxygen species levels and radioresistance in cancer stem cells. Nature.

[202] Saini N., Yang X. (2018). Metformin as an anti-cancer agent: Actions and mechanisms targeting cancer stem cells. Acta Biochim. Biophys. Sin..

[203] Wang N., Wang Z., Peng C., You J., Shen J., Han S., Chen J. (2014). Dietary compound isoliquiritigenin targets GRP78 to chemosensitize breast cancer stem cells via β-catenin/ABCG2 signaling. Carcinogenesis.

[204] Mao J., Song B., Shi Y., Wang B., Fan S., Yu X., Tang J., Li L. (2013). ShRNA targeting Notch1 sensitizes breast cancer stem cell to paclitaxel. Int. J. Biochem. Cell Biol..

[205] Jia D., Tan Y., Liu H., Ooi S., Li L., Wright K., Bennett S., Addison C.L., Wang L. (2016). Cardamonin reduces chemotherapy-enriched breast cancer stem-like cells in vitro and in vivo. Oncotarget.

[206] Saha S., Mukherjee S., Khan P., Kajal K., Mazumdar M., Manna A., Mukherjee S., De S., Jana D., Sarkar D.K. (2016). Aspirin Suppresses the Acquisition of Chemoresistance in Breast Cancer by Disrupting an NFκB–IL6 Signaling Axis Responsible for the Generation of Cancer Stem Cells. Cancer Res..

[207] Woo Y., Oh J., Kim J.-S. (2017). Suppression of Nrf2 Activity by Chestnut Leaf Extract Increases Chemosensitivity of Breast Cancer Stem Cells to Paclitaxel. Nutrients.

[208] Pandrangi S.L., Chikati R., Chauhan P.S., Kumar C.S., Banarji A., Saxena S. (2014). Effects of ellipticine on ALDH1A1-expressing breast cancer stem cells—an in vitro and in silico study. Tumor Biol..

[209] Samanta D., Park Y., Andrabi S.A., Shelton L.M., Gilkes D.M., Semenza G.L. (2016). PHGDH Expression Is Required for Mitochondrial Redox Homeostasis, Breast Cancer Stem Cell Maintenance, and Lung Metastasis. Cancer Res..

[210] Sims-Mourtada J., Opdenaker L.M., Davis J., Arnold K.M., Flynn D. (2015). Taxane-induced hedgehog signaling is linked to expansion of breast cancer stem-like populations after chemotherapy. Mol. Carcinog..

[211] Wang X., Wang X., Gu J., Zhou M., He Z., Wang X., Ferrone S. (2017). Overexpression of miR-489 enhances efficacy of 5-fluorouracil-based treatment in breast cancer stem cells by targeting XIAP. Oncotarget.

[212] Xie Q., Wang S., Zhao Y., Zhang Z., Qin C., Yang X. (2017). MiR-519d impedes cisplatin-resistance in breast cancer stem cells by down-regulating the expression of MCL-1. Oncotarget.

[213] Korkaya H., Kim G.-i., Davis A., Malik F., Henry N.L., Ithimakin S., Quraishi A.A., Tawakkol N., D’Angelo R., Paulson A.K. (2012). Activation of an IL6 Inflammatory Loop Mediates Trastuzumab Resistance in HER2+ Breast Cancer by Expanding the Cancer Stem Cell Population. Mol. Cell.

[214] Achuthan S., Santhoshkumar T.R., Prabhakar J., Nair S.A., Pillai M.R. (2011). Drug-induced Senescence Generates Chemoresistant Stemlike Cells with Low Reactive Oxygen Species. J. Biol. Chem..

[215] Sun R., Liu Y., Li S.-Y., Shen S., Du X.-J., Xu C.-F., Cao Z.-T., Bao Y., Zhu Y.-H., Li Y.-P. (2015). Co-delivery of all-trans-retinoic acid and doxorubicin for cancer therapy with synergistic inhibition of cancer stem cells. Biomaterials.

[216] Sun R., Shen S., Zhang Y.-J., Xu C.-F., Cao Z.-T., Wen L.-P., Wang J. (2016). Nanoparticle-facilitated autophagy inhibition promotes the efficacy of chemotherapeutics against breast cancer stem cells. Biomaterials.

[217] Li S.-Y., Sun R., Wang H.-X., Shen S., Liu Y., Du X.-J., Zhu Y.-H., Jun W. (2015). Combination therapy with epigenetic-targeted and chemotherapeutic drugs delivered by nanoparticles to enhance the chemotherapy response and overcome resistance by breast cancer stem cells. J. Control. Release.

[218] Kolev V.N., Tam W.F., Wright Q.G., McDermott S.P., Vidal C.M., Shapiro I.M., Xu Q., Wicha M.S., Pachter J.A., Weaver D.T. (2017). Inhibition of FAK kinase activity preferentially targets cancer stem cells. Oncotarget.

[219] Samanta D., Gilkes D.M., Chaturvedi P., Xiang L., Semenza G.L. (2014). Hypoxia-inducible factors are required for chemotherapy resistance of breast cancer stem cells. Proc. Natl. Acad. Sci. USA.

[220] Burnett J.P., Lim G., Li Y., Shah R.B., Lim R., Paholak H.J., McDermott S.P., Sun L., Tsume Y., Bai S. (2017). Sulforaphane enhances the anticancer activity of taxanes against triple negative breast cancer by killing cancer stem cells. Cancer Lett..

[221] Ginestier C., Liu S., Diebel M.E., Korkaya H., Luo M., Brown M., Wicinski J., Cabaud O., Charafe-Jauffret E., Birnbaum D. (2010). CXCR1 blockade selectively targets human breast cancer stem cells in vitro and in xenografts. J. Clin. Investig..

[222] Kolev V.N., Wright Q.G., Vidal C.M., Ring J.E., Shapiro I.M., Ricono J., Weaver D.T., Padval M.V., Pachter J.A., Xu Q. (2015). PI3K/mTOR Dual Inhibitor VS-5584 Preferentially Targets Cancer Stem Cells. Cancer Res..

[223] Yu Q.C., Verheyen E.M., Zeng Y.A. (2016). Mammary Development and Breast Cancer: A Wnt Perspective. Cancers.

[224] Yook J.I., Li X.-Y., Ota I., Hu C., Kim H.S., Kim N.H., Cha S.Y., Ryu J.K., Choi Y.J., Kim J. (2006). A Wnt–Axin2–GSK3β cascade regulates Snail1 activity in breast cancer cells. Nat. Cell Biol..

[225] Xu J., Prosperi J.R., Choudhury N., Olopade O.I., Goss K.H. (2015). β-Catenin Is Required for the Tumorigenic Behavior of Triple-Negative Breast Cancer Cells. PLoS ONE.

[226] Harrison H., Farnie G., Howell S.J., Rock R.E., Stylianou S., Brennan K.R., Bundred N.J., Clarke R.B. (2010). Regulation of breast cancer stem cell activity by signaling through the Notch4 receptor. Cancer Res..

[227] Kim B., Stephen S.L., Hanby A.M., Horgan K., Perry S.L., Richardson J., Roundhill E.A., Valleley E.M.A., Verghese E.T., Williams B.J. (2015). Chemotherapy induces Notch1-dependent MRP1 up-regulation, inhibition of which sensitizes breast cancer cells to chemotherapy. BMC Cancer.

[228] Schott A.F., Landis M.D., Dontu G., Griffith K.A., Layman R.M., Krop I., Paskett L.A., Wong H., Dobrolecki L.E., Lewis M.T. (2013). Preclinical and Clinical Studies of Gamma Secretase Inhibitors with Docetaxel on Human Breast Tumors. Clin. Cancer Res..

[229] Mamaeva V., Niemi R., Beck M., Özliseli E., Desai D., Landor S., Gronroos T., Kronqvist P., Pettersen I.K.N., McCormack E. (2016). Inhibiting Notch Activity in Breast Cancer Stem Cells by Glucose Functionalized Nanoparticles Carrying γ-secretase Inhibitors. Mol. Ther..

[230] Park E.Y., Chang E., Lee E.J., Lee H.-W., Kang H.-G., Chun K.-H., Woo Y.M., Kong H.K., Ko J.Y., Suzuki H. (2014). Targeting of miR34a–NOTCH1 Axis Reduced Breast Cancer Stemness and Chemoresistance. Cancer Res..

[231] Habib J.G., O’Shaughnessy J.A. (2016). The hedgehog pathway in triple-negative breast cancer. Cancer Med..

[232] Li X., Deng W., Nail C.D., Bailey S.K., Kraus M.H., Ruppert J.M., Lobo-Ruppert S.M. (2006). Snail induction is an early response to Gli1 that determines the efficiency of epithelial transformation. Oncogene.

[233] Tao Y., Mao J., Zhang Q., Li L. (2011). Overexpression of Hedgehog signaling molecules and its involvement in triple-negative breast cancer. Oncol. Lett..

[234] Das S., Samant R.S., Shevde L.A. (2013). Nonclassical Activation of Hedgehog Signaling Enhances Multidrug Resistance and Makes Cancer Cells Refractory to Smoothened-targeting Hedgehog Inhibition. J. Biol. Chem..

[235] Arnold K.M., Flynn N.J., Sims-Mourtada J. (2015). Activation of Inflammatory Responses Correlate With Hedgehog Activation and Precede Expansion of Cancer Stem-Like Cells in an Animal Model of Residual Triple Negative Breast Cancer after Neoadjuvant Chemotherapy. Cancer Stud. Mol. Med..

[236] Zhou J., Wulfkuhle J., Zhang H., Gu P., Yang Y., Deng J., Margolick J.B., Liotta L.A., Petricoin E., Zhang Y. (2007). Activation of the PTEN/mTOR/STAT3 pathway in breast cancer stem-like cells is required for viability and maintenance. Proc. Natl. Acad. Sci. USA.

[237] Karthik G.-M., Ma R., Lövrot J., Kis L.L., Lindh C., Blomquist L., Fredriksson I., Bergh J., Hartman J. (2015). mTOR inhibitors counteract tamoxifen-induced activation of breast cancer stem cells. Cancer Lett..

[238] Daverey A., Drain A.P., Kidambi S. (2015). Physical Intimacy of Breast Cancer Cells with Mesenchymal Stem Cells Elicits Trastuzumab Resistance through Src Activation. Sci. Rep..

[239] Liu M., Sakamaki T., Casimiro M.C., Willmarth N.E., Quong A.A., Ju X., Ojeifo J., Jiao X., Yeow W.-S., Katiyar S. (2010). The canonical NF-kappaB pathway governs mammary tumorigenesis in transgenic mice and tumor stem cell expansion. Cancer Res..

[240] Vazquez-Santillan K., Melendez-Zajgla J., Jimenez-Hernandez L.E., Gaytan-Cervantes J., Muñoz-Galindo L., Piña-Sanchez P., Martinez-Ruiz G., Torres J., Garcia-Lopez P., Gonzalez-Torres C. (2016). NF-kappaΒ-inducing kinase regulates stem cell phenotype in breast cancer. Sci. Rep..

[241] Smith S.M., Lyu Y.L., Cai L. (2014). NF-kappaB affects proliferation and invasiveness of breast cancer cells by regulating CD44 expression. PLoS ONE.

[242] Neuzillet C., Tijeras-Raballand A., Cohen R., Cros J., Faivre S., Raymond E., de Gramont A. (2015). Targeting the TGFβ pathway for cancer therapy. Pharmacol. Ther..

[243] Wahdan-Alaswad R.S., Thor A.D., Stoian A.P., Rizzo M. (2020). Metformin Activity against Breast Cancer: Mechanistic Differences by Molecular Subtype and Metabolic Conditions. Metformin.

[244] Cufi S., Corominas-Faja B., Vazquez-Martin A., Oliveras-Ferraros C., Dorca J., Bosch-Barrera J., Martin-Castillo B., Menendez J.A. (2012). Metformin-induced preferential killing of breast cancer initiating CD44+CD24-/low cells is sufficient to overcome primary resistance to trastuzumab in HER2+ human breast cancer xenografts. Oncotarget.

[245] Hirsch H.A., Iliopoulos D., Tsichlis P.N., Struhl K. (2009). Metformin selectively targets cancer stem cells, and acts together with chemotherapy to block tumor growth and prolong remission. Cancer Res..

[246] Vazquez-Martin A., Oliveras-Ferraros C., Del Barco S., Martin-Castillo B., Menendez J.A. (2011). The anti-diabetic drug metformin suppresses self-renewal and proliferation of trastuzumab-resistant tumor-initiating breast cancer stem cells. Breast Cancer Res. Treat..

[247] Bao B., Azmi A.S., Ali S., Zaiem F., Sarkar F.H. (2014). Metformin may function as anti-cancer agent via targeting cancer stem cells: The potential biological significance of tumor-associated miRNAs in breast and pancreatic cancers. Ann. Transl. Med..

[248] Zou Y.F., Xie C.W., Yang S.X., Xiong J.P. (2017). AMPK activators suppress breast cancer cell growth by inhibiting DVL3-facilitated Wnt/β-catenin signaling pathway activity. Mol. Med. Rep..

[249] Liu X., Chhipa R.R., Nakano I., Dasgupta B. (2014). The AMPK Inhibitor Compound C Is a Potent AMPK-Independent Antiglioma Agent. Mol. Cancer Ther..

[250] Samuel S.M., Satheesh N.J., Ghosh S., Büsselberg D., Majeed Y., Ding H., Triggle C.R. (2019). Treatment with a Combination of Metformin and 2-Deoxyglucose Upregulates Thrombospondin-1 in Microvascular Endothelial Cells: Implications in Anti-Angiogenic Cancer Therapy. Cancers.

[251] Fan C., Wang Y., Liu Z., Sun Y., Wang X., Wei G., Wei J. (2015). Metformin exerts anticancer effects through the inhibition of the Sonic hedgehog signaling pathway in breast cancer. Int. J. Mol. Med..

[252] Dunphy K.A., Seo J.-H., Kim D.J., Roberts A.L., Tao L., DiRenzo J., Balboni A.L., Crisi G.M., Hagen M.J., Chandrasekaran T. (2013). Oncogenic transformation of mammary epithelial cells by transforming growth factor beta independent of mammary stem cell regulation. Cancer Cell Int..

[253] Woosley A.N., Dalton A.C., Hussey G.S., Howley B.V., Mohanty B.K., Grelet S., Dincman T., Bloos S., Olsen S.K., Howe P.H. (2019). TGFβ promotes breast cancer stem cell self-renewal through an ILEI/LIFR signaling axis. Oncogene.

[254] Konge J., Leteurtre F., Goislard M., Biard D., Morel-Altmeyer S., Vaurijoux A., Gruel G., Chevillard S., Lebeau J. (2018). Breast cancer stem cell-like cells generated during TGFβ-induced EMT are radioresistant. Oncotarget.

[255] Karicheva O., Rodriguez-Vargas J.M., Wadier N., Martin-Hernandez K., Vauchelles R., Magroun N., Tissier A., Schreiber V., Dantzer F. (2016). PARP3 controls TGFβ and ROS driven epithelial-to-mesenchymal transition and stemness by stimulating a TG2-Snail-E-cadherin axis. Oncotarget.

[256] Wahdan-Alaswad R., Harrell J.C., Fan Z., Edgerton S.M., Liu B., Thor A.D. (2016). Metformin attenuates transforming growth factor beta (TGF-β) mediated oncogenesis in mesenchymal stem-like/claudin-low triple negative breast cancer. Cell Cycle.

[257] Leonel C., Borin T.F., de Carvalho Ferreira L., Moschetta M.G., Bajgelman M.C., Viloria-Petit A.M., de Campos Zuccari D.A.P. (2017). Inhibition of Epithelial-Mesenchymal Transition and Metastasis by Combined TGFbeta Knockdown and Metformin Treatment in a Canine Mammary Cancer Xenograft Model. J. Mammary Gland Biol. Neoplasia.

[258] Vazquez-Martin A., Oliveras-Ferraros C., Cufí S., Del Barco S., Martin-Castillo B., Menendez J.A. (2010). Metformin regulates breast cancer stem cell ontogeny by transcriptional regulation of the epithelial-mesenchymal transition (EMT) status. Cell Cycle.

[259] Zhao M., Wang Y., Du C., Liu Y., Zhang N., Luo F. (2018). Aspirin and metformin exhibit antitumor activity in murine breast cancer. Oncol. Rep..

[260] Iliopoulos D., Hirsch H.A., Struhl K. (2009). An epigenetic switch involving NF-kappaB, Lin28, Let-7 MicroRNA, and IL6 links inflammation to cell transformation. Cell.

[261] Hirsch H.A., Iliopoulos D., Struhl K. (2013). Metformin inhibits the inflammatory response associated with cellular transformation and cancer stem cell growth. Proc. Natl. Acad. Sci. USA.

[262] Zhao W., Zhang X., Liu J., Sun B., Tang H., Zhang H. (2016). miR-27a-mediated antiproliferative effects of metformin on the breast cancer cell line MCF-7. Oncol. Rep..

[263] Truong Do M., Gyun Kim H., Ho Choi J., Gwang Jeong H. (2014). Metformin induces microRNA-34a to downregulate the Sirt1/Pgc-1α/Nrf2 pathway, leading to increased susceptibility of wild-type p53 cancer cells to oxidative stress and therapeutic agents. Free Radic. Biol. Med..

[264] Oliveras-Ferraros C., Cufí S., Vazquez-Martin A., Torres-Garcia V.Z., Del Barco S., Martin-Castillo B., Menendez J.A. (2011). Micro(mi)RNA expression profile of breast cancer epithelial cells treated with the anti-diabetic drug metformin: Induction of the tumor suppressor miRNA let-7a and suppression of the TGFβ-induced oncomiR miRNA-181a. Cell Cycle.

[265] Wahdan-Alaswad R.S., Cochrane D.R., Spoelstra N.S., Howe E.N., Edgerton S.M., Anderson S.M., Thor A.D., Richer J.K. (2014). Metformin-induced killing of triple-negative breast cancer cells is mediated by reduction in fatty acid synthase via miRNA-193b. Horm Cancer.

[266] Feng F., Zhang J., Fan X., Yuan F., Jiang Y., Lv R., Ma Y. (2017). Downregulation of Rab27A contributes to metformin-induced suppression of breast cancer stem cells. Oncol. Lett..

[267] Janzer A., German N.J., Gonzalez-Herrera K.N., Asara J.M., Haigis M.C., Struhl K. (2014). Metformin and phenformin deplete tricarboxylic acid cycle and glycolytic intermediates during cell transformation and NTPs in cancer stem cells. Proc. Natl. Acad. Sci. USA.

[268] Brown S.L., Kolozsvary A., Isrow D.M., Al Feghali K., Lapanowski K., Jenrow K.A., Kim J.H. (2019). A Novel Mechanism of High Dose Radiation Sensitization by Metformin. Front. Oncol..

[269] Chan D.K., Miskimins W.K. (2012). Metformin and phenethyl isothiocyanate combined treatment in vitro is cytotoxic to ovarian cancer cultures. J. Ovarian Res..

[270] Christensen M.M., Hojlund K., Hother-Nielsen O., Stage T.B., Damkier P., Beck-Nielsen H., Brosen K. (2015). Steady-state pharmacokinetics of metformin is independent of the OCT1 genotype in healthy volunteers. Eur. J. Clin. Pharm..

[271] Kinaan M., Ding H., Triggle C.R. (2015). Metformin: An Old Drug for the Treatment of Diabetes but a New Drug for the Protection of the Endothelium. Med. Princ. Pract..

[272] Lee H., Park H.J., Park C.-S., Oh E.-T., Choi B.-H., Williams B., Lee C.K., Song C.W. (2014). Response of Breast Cancer Cells and Cancer Stem Cells to Metformin and Hyperthermia Alone or Combined. PLoS ONE.

[273] Shi P., Liu W., Tala, Wang H., Li F., Zhang H., Wu Y., Kong Y., Zhou Z., Wang C. (2017). Metformin suppresses triple-negative breast cancer stem cells by targeting KLF5 for degradation. Cell Discov..

[274] Cuyàs E., Martin-Castillo B., Bosch-Barrera J., Menendez J.A. (2017). Metformin inhibits RANKL and sensitizes cancer stem cells to denosumab. Cell Cycle.

[275] Tan W., Tang H., Jiang X., Ye F., Huang L., Shi D., Li L., Huang X., Li L., Xie X. (2019). Metformin mediates induction of miR-708 to inhibit self-renewal and chemoresistance of breast cancer stem cells through targeting CD47. J. Cell. Mol. Med..

[276] Song C.W., Lee H., Dings R.P.M., Williams B., Powers J., Santos T.D., Choi B.-H., Park H.J. (2012). Metformin kills and radiosensitizes cancer cells and preferentially kills cancer stem cells. Sci. Rep..

[277] Kim T.H., Suh D.H., Kim M.-K., Song Y.S. (2014). Metformin against Cancer Stem Cells through the Modulation of Energy Metabolism: Special Considerations on Ovarian Cancer. BioMed Res. Int..

[278] Iliopoulos D., Hirsch H.A., Struhl K. (2011). Metformin decreases the dose of chemotherapy for prolonging tumor remission in mouse xenografts involving multiple cancer cell types. Cancer Res..

[279] Bednar F., Simeone D.M. (2012). Metformin and cancer stem cells: Old drug, new targets. Cancer Prev. Res..

[280] Rattan R., Ali Fehmi R., Munkarah A. (2012). Metformin: An emerging new therapeutic option for targeting cancer stem cells and metastasis. J. Oncol..

[281] Aponte P.M., Caicedo A. (2017). Stemness in Cancer: Stem Cells, Cancer Stem Cells, and Their Microenvironment. Stem Cells Int..

[282] Bozic I., Reiter J.G., Allen B., Antal T., Chatterjee K., Shah P., Moon Y.S., Yaqubie A., Kelly N., Le D.T. (2013). Evolutionary dynamics of cancer in response to targeted combination therapy. eLife.

[283] Banerjee A., Birts C.N., Darley M., Parker R., Mirnezami A.H., West J., Cutress R.I., Beers S.A., Rose-Zerilli M.J.J., Blaydes J.P. (2019). Stem cell-like breast cancer cells with acquired resistance to metformin are sensitive to inhibitors of NADH-dependent CtBP dimerization. Carcinogenesis.

[284] Oliveras-Ferraros C., Vazquez-Martin A., Cuyàs E., Corominas-Faja B., Rodríguez-Gallego E., Fernández-Arroyo S., Martin-Castillo B., Joven J., Menendez Menendez J. (2014). Acquired resistance to metformin in breast cancer cells triggers transcriptome reprogramming toward a degradome-related metastatic stem-like profile. Cell Cycle.

[285] Scherbakov A.M., Sorokin D.V., Tatarskiy Jr V.V., Prokhorov N.S., Semina S.E., Berstein L.M., Krasil’nikov M.A. (2016). The phenomenon of acquired resistance to metformin in breast cancer cells: The interaction of growth pathways and estrogen receptor signaling. IUBMB Life.

[286] Menendez J.A., Oliveras-Ferraros C., Cufi S., Corominas-Faja B., Joven J., Martin-Castillo B., Vazquez-Martin A. (2012). Metformin is synthetically lethal with glucose withdrawal in cancer cells. Cell Cycle.

[287] Zordoky B.N., Bark D., Soltys C.L., Sung M.M., Dyck J.R. (2014). The anti-proliferative effect of metformin in triple-negative MDA-MB-231 breast cancer cells is highly dependent on glucose concentration: Implications for cancer therapy and prevention. Biochim. Biophys. Acta.

[288] Snima K.S., Jayakumar R., Unnikrishnan A.G., Nair S.V., Lakshmanan V.-K. (2012). O-Carboxymethyl chitosan nanoparticles for metformin delivery to pancreatic cancer cells. Carbohydr. Polym..

[289] Graham G.G., Punt J., Arora M., Day R.O., Doogue M.P., Duong J., Furlong T.J., Greenfield J.R., Greenup L.C., Kirkpatrick C.M. (2011). Clinical Pharmacokinetics of Metformin. Clin. Pharmacokinet..

[290] Cetin M., Sahin S. (2016). Microparticulate and nanoparticulate drug delivery systems for metformin hydrochloride. Drug Deliv..

[291] Javidfar S., Pilehvar-Soltanahmadi Y., Farajzadeh R., Lotfi-Attari J., Shafiei-Irannejad V., Hashemi M., Zarghami N. (2018). The inhibitory effects of nano-encapsulated metformin on growth and hTERT expression in breast cancer cells. J. Drug Deliv. Sci. Technol..

[292] Baldassari S., Solari A., Zuccari G., Drava G., Pastorino S., Fucile C., Marini V., Daga A., Pattarozzi A., Ratto A. (2018). Development of an Injectable Slow-Release Metformin Formulation and Evaluation of Its Potential Antitumor Effects. Sci. Rep..

[293] De Jong W.H., Borm P.J.A. (2008). Drug delivery and nanoparticles:applications and hazards. Int. J. Nanomed..

[294] Schmid P., Adams S., Rugo H.S., Schneeweiss A., Barrios C.H., Iwata H., Dié V., Hegg R., Im S.-A., Shaw Wright G. (2018). Atezolizumab and Nab-Paclitaxel in Advanced Triple-Negative Breast Cancer. N. Engl. J. Med..

[295] Kuwayama T., Nakamura S., Hayashi N., Takano T., Tsugawa K., Sato T., Kitani A., Okuyama H., Yamauchi H. (2018). Randomized Multicenter Phase II Trial of Neoadjuvant Therapy Comparing Weekly Nab-paclitaxel Followed by FEC With Docetaxel Followed by FEC in HER2^−^ Early-stage Breast Cancer. Clin. Breast Cancer.

[296] Farajzadeh R., Pilehvar-Soltanahmadi Y., Dadashpour M., Javidfar S., Lotfi-Attari J., Sadeghzadeh H., Shafiei-Irannejad V., Zarghami N. (2018). Nano-encapsulated metformin-curcumin in PLGA/PEG inhibits synergistically growth and hTERT gene expression in human breast cancer cells. Artif. Cells Nanomed. Biotechnol..

[297] Shukla S.K., Kulkarni N.S., Chan A., Parvathaneni V., Farrales P., Muth A., Gupta V. (2019). Metformin-Encapsulated Liposome Delivery System: An Effective Treatment Approach against Breast Cancer. Pharmaceutics.

[298] Aljofan M., Riethmacher D. (2019). Anticancer activity of metformin: A systematic review of the literature. Future Sci. OA.

[299] DeVito N.C., Goodman M.D., Caplain J., Rajagopal S., Popowich D., Orkin B.A., Grimm E., Tsichlis P.N., Martell R.E., Saif W.M. (2014). A pilot study evaluating the safety and impact of pretreatment with metformin on colorectal cancer stem cells (CCSC) in patients undergoing resection. J. Clin. Oncol..

[300] Brown J.R., Chan D.K., Shank J.J., Griffith K.A., Fan H., Szulawski R., Yang K., Reynolds R.K., Johnston C., McLean K. (2020). Phase II clinical trial of metformin as a cancer stem cell-targeting agent in ovarian cancer. JCI Insight.

